# New insights of the correlation between *AXIN2* polymorphism and cancer risk and susceptibility: evidence from 72 studies

**DOI:** 10.1186/s12885-021-08092-0

**Published:** 2021-04-01

**Authors:** Xi Li, Yiming Li, Guodong Liu, Wei Wu

**Affiliations:** 1 Department of Geriatric Surgery, Xiangya Hospital, Central South University, Changsha, 410008 China; 2 National Clinical Research Center for Geriatric Disorders, Xiangya Hospital, Central South University, Changsha, 410008 China; 3 Department of General Surgery, Xiangya Hospital, Central South University, Changsha, 410008 China

**Keywords:** *AXIN2*, Polymorphism, Cancer, Analysis, Correlation

## Abstract

**Background:**

Numerous studies have reported the correlation between *AXIN2* polymorphism and cancer risk, but the results seem not consistent. In order to get an overall, accurate and updated results about *AXIN2* polymorphism and cancer risk, we conducted this study.

**Methods:**

An updated analysis was performed to analyze the correlation between *AXIN2* polymorphisms and cancer risk. Linkage disequilibrium (LD) analysis was also used to show the associations.

**Results:**

Seventy-two case-control studies were involved in the study, including 22,087 cases and 18,846 controls. The overall results showed *rs11079571* had significant association with cancer risk (allele contrast model: OR = 0.539, 95%CI = 0.478–0.609, PAdjust = 0.025; homozygote model: OR = 0.22, 95% CI = 0.164–0.295, PAdjust< 0.001; heterozygote model: OR = 0.292, 95% CI = 0.216–0.394, PAdjust< 0.001; dominant model: OR = 0.249, 95% CI = 0.189–0.33, PAdjust< 0.001). The same results were obtained with *rs1133683* in homozygote and recessive models (PAdjust< 0.05), and in *rs35285779* in heterozygote and dominant models (PAdjust< 0.05). LD analysis revealed significant correlation between *rs7210356* and *rs9915936* in the populations of CEU, CHB&CHS, ESN and JPT (CEU: *r*^2^ = 0.91; CHB&CHS: *r*^2^ = 0.74; ESN: *r*^2^ = 0.62, JPT: *r*^2^ = 0.57), and a significant correlation between *rs9915936* and *rs7224837* in the populations of CHB&CHS, ESN and JPT (*r*^2^>0.5), between *rs7224837* and *rs7210356* in the populations of CEU, CHB&CHS, JPT (*r*^2^>0.5), between *rs35435678* and *rs35285779* in the populations of CEU, CHB&CHS and JPT (*r*^2^>0.5).

**Conclusions:**

*AXIN2 rs11079571, rs1133683* and *rs35285779* polymorphisms have significant correlations with overall cancer risk. What’s more, two or more polymorphisms such as *rs7210356* and *rs9915936, rs9915936* and *rs7224837, rs7224837* and *rs7210356, rs35435678* and *rs35285779* have significant correlation with cancer susceptibility in different populations.

**Supplementary Information:**

The online version contains supplementary material available at 10.1186/s12885-021-08092-0.

## Background

Cancer is currently one of the most important health problems across the world, and it has been well known as the second most common cause of death in the US. According to reports, the estimated data of Cancer Statistics show that 1,762,450 new cases of cancers will be diagnosed in the US in 2019, and 606,880 deaths will be confirmed [1]. Among which, prostate cancer, lung cancer, bronchus cancer and colorectal cancer will account for the top 4 common types in male cases, and breast, lung and colorectal cancers will be the top 3 most common types in female cases [1]. The data from National Central Cancer Registry of China reported that in 2015, 4292,000 new cancer cases and 2814,000 cancer deaths occurred in China, with lung cancer being the most common incident cancer and the leading cause of cancer death. Stomach, esophageal, and liver cancers were also commonly diagnosed and were identified as leading causes of cancer death [2]. In Europe, there were an estimated 3.91 million new cases of cancer and 1.93 million deaths from cancer in 2018, among which, the female breast, colorectal, lung and prostate cancer were the most common cancer sites [3]. In recent years, many studies have pointed out that genomic types may be closely related to the carcinogenic effects of cancers, one of which is the Axin-related protein, *AXIN2* [4–7].

The *AXIN2* gene locates at chromosome 17q23–24, which belongs to a heterozygosity region that frequently loss in neuroblastoma, breast cancer, and other cancers [8, 9]. For the biological function, *AXIN2* is a critical regulator in Wnt/β-catenin signaling, especially for the stability of β-catenin, which plays an important role in cell growth, genesis of a number of malignancies, tumor progression and so on. For example, Chen et al. [10] reported that miR-183 could regulate bladder cancer cells growth and apoptosis via targeting *AXIN2*. A recent report by Chen et al. pointed out that down regulating *AXIN2* expression could promote human osteosarcoma cell proliferation [11]. Another paper showed that targeting *AXIN2* axis could suppress tumor growth and metastasis in colorectal cancer [12]. As the expression or protein structure may be influenced by gene polymorphism, some studies have taken insights in the correlation between *AXIN2* and cancer susceptibility. Otero L et al. reported that rs2240308 polymorphism was associated with colorectal cancer (CRC) and the CRC patients who carried this variation in the AXIN2 gene always had a worse prognosis [13]. Zhong et al. showed that the Axin2–148 C/T polymorphism was significantly associated with a decreased risk of cancer, particularly lung cancer, in Asians and population-based controls [14]. Liu et al. showed that rs11655966, rs3923086 and rs7591 of AXIN2 showed significant associations with papillary thyroid carcinoma (PTC) [15]. However the available results remain inconsistent. For example, E•Pinarbasi et al. [16] reported that *rs2240308* polymorphism had no significant correlation with the susceptibility of prostate cancer in the Turkish population, whereas Xu et al. [17] revealed that *AXIN2 rs2240308* variants may be associated with decreased cancer susceptibility. At the same time, Dai et al. [18] concluded that *AXIN2 rs2240308* polymorphism might decrease the susceptibility of lung and prostate cancers. Thus, we designed this meta-analysis to obtain updated and accurate insight to assess the association between *AXIN2* polymorphism and cancer susceptibility.

## Methods

### Literature retrieval strategy and eligibility criteria

Wanfang, CNKI, CBM, EMBASE, Web of Science and PubMed databases were used to search the published papers before July, 2020 by using the keywords and MeSH terms of ‘Axin OR *AXIN-2*’ AND ‘carcinoma OR cancer OR tumor’ AND ‘SNP OR mutation OR polymorphism OR variant’. All publications in English and Chinese were involved, references were also evaluated manually to get more comprehensive studies.

The studies that met the following criteria would be included: (1) case-control studies that were related to the correlation of *AXIN-2* polymorphism and cancer susceptibility; (2) English or Chinese publications, and (3) genotype frequency were provided directly or indirectly. Conversely, the studies that met the following criteria would be excluded: (1) meta-analysis, reviews, case reports or duplicate publications; (2) data of genotype frequency was not informed; (3) data from cell lines or animals.

### Data extraction

All data were examined by two independent researchers (Li X and Li YM). From which, the first author’s name, published data, total number of participants, subtypes like cancer type, source of control and ethnicity, genotyping method, and genotype frequency of the *AXIN2* gene polymorphisms in all cases and controls were labeled and calculated. Any disagreement would be re-examined and discussed by the other researchers (Liu G and Wu W) and, if necessary, the author of the publications would be requested to provide more data.

### Statistical analysis

In our study, we used five genetic models to evaluate the correlation of *AXIN2* gene polymorphisms and cancer risk, including allele contrast model (B vs. A), homozygote comparison model (BB vs. AA), heterozygote comparison model (BA vs. AA), dominant comparison model (BB + BA vs. AA), and recessive comparison model (BB vs. BA+AA). The strength of the association was checked by OR with 95% CI, and the significant statistics was confirmed by Z-test and adjusted by Bonferroni corrections, *P*_*Adjust*_ = *P*_*z*_ * 5 genetic models [19]. Subtypes like ethnicity, type of cancer and source of control were also evaluated by stratified analysis. The χ2-test was assessed to analyze the heterogeneity between studies.

*P* < 0.1 meant a significant heterogeneity, and if so, we used the random effects model (DerSimonian and Laird methods) to summarize the data [20]; if not, the fixed effect model (Mantel-Haenszel method) was selected [21]. Hardy–Weinberg equilibrium (HWE) was performed for sensitivity analysis [22]. Begg’s funnel plots and Egger’s line regression test [23, 24] were performed to assess the potential publication bias. STATA software system v12.0 was used to perform statistical analysis. *P* ≤ 0.05 was considered as a statistically significant difference.

### Linkage disequilibrium (LD) analysis

The data was acquired from 1000 Genomes Project which contains *AXIN2* polymorphisms in the present research. Six groups including CEU (Utah residents with Northern and Western European ancestry from the CEPH collection), CHS (southern Han Chinese, China), CHB (Han Chinese in Beijing, China), ESN (Esan in Nigeria), YRI (Yoruba in Ibadan, Nigeria) and JPT (Japanese in Tokyo, Japan) were involved in the program. Haploview software was performed to analyze the data, and LD analysis was performed by r^2^ statistics.

## Results

### Details of included studies

**Fig. 1:**
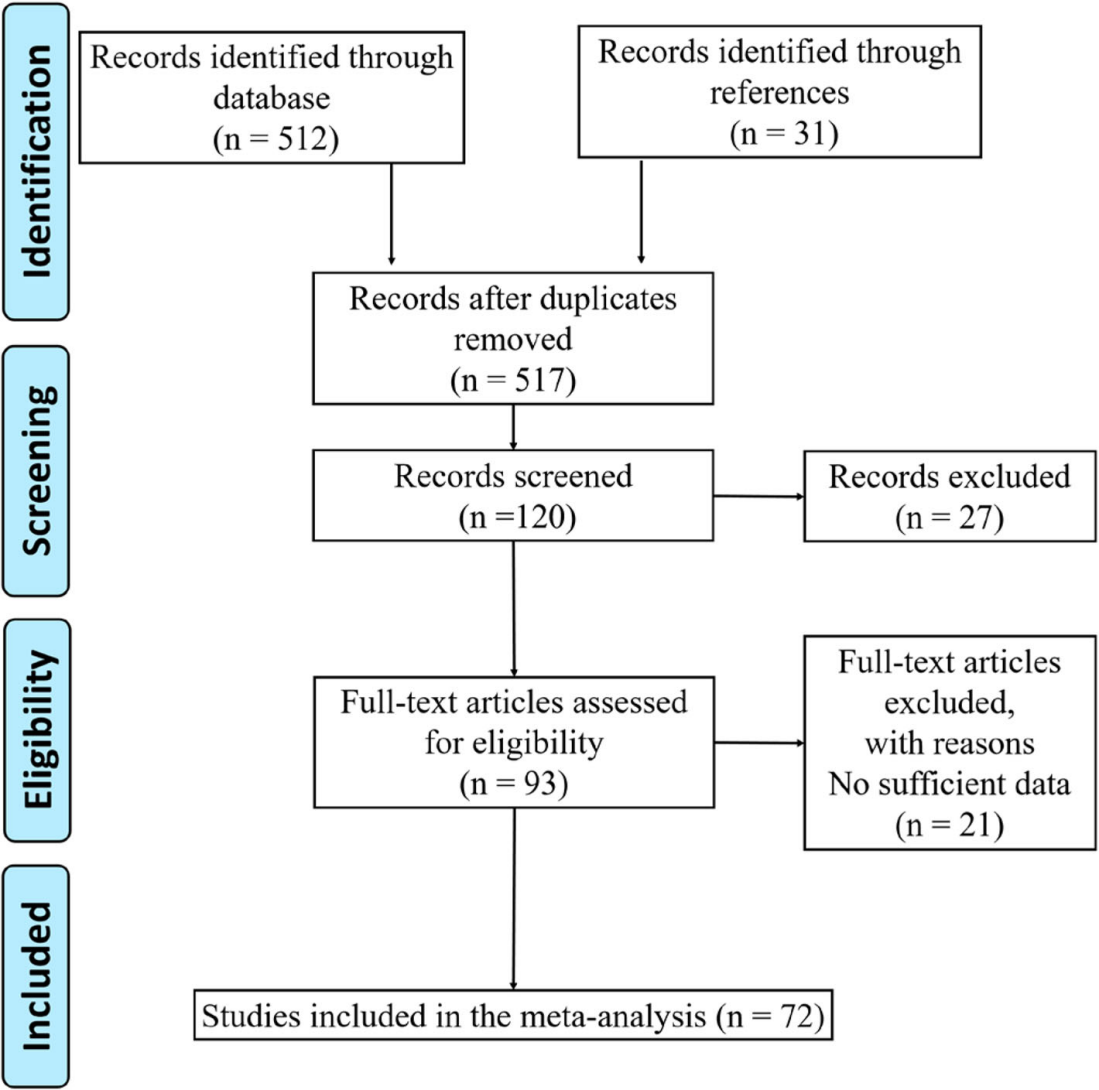
Flow chart of select methods of the study

Totally, 24 articles were included in this analysis, which contained 72 case-control studies (Fig. 1). Among which, three studies related to the linkage between *rs11079571* polymorphism and cancer susceptibility [25–27], six studies focused on *rs1133683* [16, 28–32], six studies concerned about *rs2240307* [16, 28–30, 33, 34]*,* 20 studies focused on *rs2240308* [15, 16, 28–30, 32–44], four studies focused on *rs35285779* [16, 28–30]*,* four studies focused on *rs35415678* [16, 28–30], five studies focused on *rs3923086* [15, 25, 26, 34, 45]*,* five studies focused on *rs3923087* [25, 26, 34, 41, 45]*,* three studies focused on *rs4072245* [16, 28, 30], five studies focused on *rs4791171* [25, 26, 34, 43, 45], four studies focused on *rs7219582* [16, 28–30]*,* three studies focused on *rs7224837* [34, 41, 46], four studies focused on *rs9915936* [16, 28–30]. Table 1 showed all details of the involved studies. Newcastle-Ottawa Scale (NOS) [40] was performed to assess the quality of each included study, and the results were showed in Table S1.

**Table 1:** Characteristics of the enrolled studies on *AXIN2* Polymorphism and cancer

Polymorphism	First author	Year	Ethnicity	Genotyping Method	Source of Control	Cancer Type	Cases			Controls			
							PAA	PAB	PBB	HAA	HAB	HBB	HWE
rs11079571	Wang et al.	2008	Caucasion	GoldenGate	PB	Breast Cancer	32	233	533	16	221	606	Y
rs11079571	Alanazi et al.	2013	Asian	TaqMan	PB	Breast Cancer	182	194	55	11	37	45	Y
rs11079571	Zhang et al.	2015	Asian	PCR	PB	Acute Leukemia	196	180	201	42	170	189	Y
rs1133683	Gunes et al.	2009	Asian	PCR	PB	Lung Cancer	172	204	10	42	50	8	Y
rs1133683	Pinarbasi et al.	2010	Asian	PCR	HB	Prostate Cancer	724	872	6	44	48	8	Y
rs1133683	Gunes et al.	2010	Asian	PCR	HB	Astrocytoma	70	306	20	42	50	8	Y
rs1133683	Davoodi et al.	2015	Asian	PCR-RFLP	PB	Ovarian Cancer	386	1210	6	58	34	8	Y
rs1133683	Rosales-Reynoso et al.	2016	Caucasion	PCR-RFLP	PB	Colorectal Cancer	124	252	19	22	57	21	Y
rs1133683	Bahl et al.	2017	Asian	PCR-RFLP	PB	Lung Cancer	190	1406	37	103	169	33	N
rs2240307	Gunes et al.	2009	Asian	PCR	PB	Lung Cancer	96	4	0	95	5	0	Y
rs2240307	Pinarbasi et al.	2010	Asian	PCR	HB	Prostate Cancer	81	3	0	98	2	0	Y
rs2240307	Gunes et al.	2010	Asian	PCR	HB	Astrocytoma	93	7	0	95	5	0	Y
rs2240307	Filho et al.	2011	Caucasion	TaqMan	HB	Oral Cancer	PA = 182	PB = 194	HA = 212		HB = 238		NA
rs2240307	Han et al.	2016	Asian	PCR	PB	Lung Cancer	63	27	12	79	36	5	Y
rs2240307	Bahl et al.	2017	Asian	PCR-RFLP	PB	Lung Cancer	342	34	0	289	16	0	Y
rs2240308	Kanzaki et al.	2006	Asian	PCR-RFLP	PB	Colorectal Cancer	54	44	15	42	52	15	Y
rs2240308	Kanzaki et al.	2006	Asian	PCR-RFLP	PB	Head and neck Cancer	25	29	9	42	52	15	Y
rs2240308	Kanzaki et al.	2006	Asian	PCR-RFLP	PB	Lung Cancer	81	71	8	42	52	15	Y
rs2240308	Gunes et al.	2009	Asian	PCR	PB	Lung Cancer	45	47	8	32	52	16	Y
rs2240308	Gunes et al.	2010	Asian	PCR	HB	Astrocytoma	39	45	16	32	52	16	Y
rs2240308	Ferna’ndez-Rozadilla et al.	2010	Caucasion	MassARRAY	HB	Colorectal Cancer	252	423	168	290	442	152	Y
rs2240308	Pinarbasi et al.	2010	Asian	PCR	HB	Prostate Cancer	30	35	19	34	48	18	Y
rs2240308	Naghibalhossaini et al.	2011	Asian	PCR-RFLP	PB	Colorectal Cancer	34	57	19	55	98	26	Y
rs2240308	Filho et al.	2011	Caucasion	TaqMan	HB	Oral Cancer	PA = 196	PB = 180	HA = 226		HB = 226		NA
rs2240308	Mostowska et al.	2013	Caucasion	PCR-RFLP	HB	Ovarian Cancer	67	115	46	71	146	65	Y
rs2240308	Liu et al.	2014	Asian	PCR	PB	Lung Cancer	235	216	47	211	255	67	Y
rs2240308	Ma et al.	2014	Asian	PCR	HB	Prostate Cancer	61	31	11	39	52	9	Y
rs2240308	Aristizabal-Pachon et al.	2015	Caucasion	PCR-RFLP	PB	Breast Cancer	20	58	24	44	55	3	N
rs2240308	Yadav et al.	2015	Asian	PCR-RFLP	PB	Gallbladder Cancer	98	108	44	192	253	119	N
rs2240308	Rosales-Reynoso et al.	2016	Caucasion	PCR-RFLP	PB	Colorectal Cancer	25	109	54	22	59	18	Y
rs2240308	Kim et al.	2016	Asian	GoldenGate	HB	Hepatocellular Carcinoma	124	100	18	246	195	41	Y
rs2240308	Han et al.	2016	Asian	PCR	PB	Lung Cancer	50	34	18	67	43	10	Y
rs2240308	Kim et al.	2016	Asian	Dynamic 96.96 ArrayTM Assay	PB	Lung Cancer	169	142	47	562	436	124	N
rs2240308	Liu et al.	2016	Asian	MassARRAY	HB	Papillary Thyroid Carcinoma	27	24	2	17	29	4	Y
rs2240308	Bahl et al.	2017	Asian	PCR-RFLP	PB	Lung Cancer	99	150	54	81	144	80	Y
rs35285779	Gunes et al.	2009	Asian	PCR	PB	Lung Cancer	77	20	3	64	28	8	Y
rs35285779	Pinarbasi et al.	2010	Asian	PCR	HB	Prostate Cancer	69	15	0	61	32	7	Y
rs35285779	Gunes et al.	2010	Asian	PCR	HB	Astrocytoma	70	25	5	64	28	8	Y
rs35285779	Bahl et al.	2017	Asian	PCR-RFLP	PB	Lung Cancer	255	46	2	248	55	2	Y
rs35415678	Gunes et al.	2009	Asian	PCR	PB	Lung Cancer	91	9	0	86	14	0	Y
rs35415678	Pinarbasi et al.	2010	Asian	PCR	HB	Prostate Cancer	83	1	0	99	1	0	Y
rs35415678	Gunes et al.	2010	Asian	PCR	HB	Astrocytoma	87	13	0	86	14	0	Y
rs35415678	Bahl et al.	2017	Asian	PCR-RFLP	PB	Lung Cancer	257	46	0	261	44	0	Y
rs3923086	Wang et al.	2008	Caucasion	GoldenGate	PB	Breast Cancer	238	395	164	284	419	139	Y
rs3923086	Filho et al.	2011	Caucasion	TaqMan	HB	Oral Cancer	PA = 172	PB = 204	HA = 212		HB = 238		NA
rs3923086	Alanazi et al.	2013	Asian	TaqMan	PB	Breast Cancer	27	41	31	16	42	35	Y
rs3923086	Liu et al.	2016	Asian	MassARRAY	HB	Papillary Thyroid Carcinoma	47	8	0	34	15	1	Y
rs3923086	Parine et al.	2019	Asian	TaqMan	PB	Colorectal Cancer	48	52	21	41	50	19	Y
rs3923087	Wang et al.	2008	Caucasion	GoldenGate	PB	Breast Cancer	47	292	458	39	278	525	Y
rs3923087	Filho et al.	2011	Caucasion	TaqMan	HB	Oral Cancer	PA = 70	PB = 306	HA = 130		HB = 320		NA
rs3923087	Mostowska et al.	2013	Caucasion	PCR-RFLP	HB	Ovarian Cancer	10	84	133	14	97	171	Y
rs3923087	Alanazi et al.	2013	Asian	TaqMan	PB	Breast Cancer	45	35	18	24	50	19	Y
rs3923087	Parine et al.	2019	Asian	TaqMan	PB	Colorectal Cancer	35	56	32	37	50	23	Y
rs4072245	Gunes et al.	2009	Asian	PCR	PB	Lung Cancer	73	27	0	80	20	0	Y
rs4072245	Pinarbasi et al.	2010	Asian	PCR	HB	Prostate Cancer	73	11	0	78	22	0	Y
rs4072245	Gunes et al.	2010	Asian	PCR	HB	Astrocytoma	82	18	0	80	20	0	Y
rs4791171	Wang et al.	2008	Caucasion	GoldenGate	PB	Breast Cancer	83	332	383	61	349	433	Y
rs4791171	Filho et al.	2011	Caucasion	TaqMan	HB	Oral Cancer	PA = 124	PB = 252	HA = 136		HB = 316		NA
rs4791171	Alanazi et al.	2013	Asian	TaqMan	PB	Breast Cancer	34	44	21	22	44	17	Y
rs4791171	Yadav et al.	2015	Asian	PCR-RFLP	PB	Gallbladder Cancer	35	118	97	88	248	228	Y
rs4791171	Parine et al.	2019	Asian	TaqMan	PB	Colorectal Cancer	40	55	27	38	48	24	Y
rs7219582	Gunes et al.	2009	Asian	PCR	PB	Lung Cancer	97	3	0	96	4	0	Y
rs7219582	Pinarbasi et al.	2010	Asian	PCR	HB	Prostate Cancer	81	3	0	95	5	0	Y
rs7219582	Gunes et al.	2010	Asian	PCR	HB	Astrocytoma	91	9	0	96	4	0	Y
rs7219582	Bahl et al.	2017	Asian	PCR-RFLP	PB	Lung Cancer	87	205	11	42	263	0	N
rs7224837	Filho et al.	2011	Caucasion	TaqMan	HB	Oral Cancer	342	34		400	50		NA
rs7224837	Mostowska et al.	2013	Caucasion	PCR-RFLP	HB	Ovarian Cancer	161	61	6	203	71	8	Y
rs7224837	Jeanne et al.	2015	Caucasion	iSelect genotyping array	HB	Bladder Cancer	646	151	6	616	169	17	Y
rs9915936	Gunes et al.	2009	Asian	PCR	PB	Lung Cancer	91	9	0	88	12	0	Y
rs9915936	Pinarbasi et al.	2010	Asian	PCR	HB	Prostate Cancer	77	7	0	92	8	0	Y
rs9915936	Gunes et al.	2010	Asian	PCR	HB	Astrocytoma	91	9	0	88	12	0	Y
rs9915936	Bahl et al.	2017	Asian	PCR-RFLP	PB	Lung Cancer	268	29	6	249	51	5	Y

*HB* Hospital Based, *PB* Population Based, *HWE* Hardy Weinberg Equilibrium, *Y* polymorphisms conformed to HWE in the control group, *N* polymorphisms didn’t conform to HWE in the control group, *NA* not available

### *AXIN-2* polymorphism and risk of cancers

Thirteen polymorphisms of *AXIN-2* were analyzed in the study. For *rs11079571* polymorphism, two studies were related to breast cancer and another was involved in acute leukemia. Among which, two were about Asian population and one was based on Caucasian. The sources of all three controls were population based. All of the three genotype distributions of controls of *rs11079571* studies were conformed to HWE, For the *rs1133683* polymorphism, six studies met the criteria, including two lung cancers and one prostate cancer, astrocytoma, ovarian cancer and colorectal cancer, respectively. Among them, five studies related to Asian and one study concerned about Caucasion population. As to *rs2240307* polymorphism, six studies were involved, three of them were about lung cancer, and the other three were about oral cancer, prostate cancer, astrocytoma, respectively. For the *rs2240308* polymorphism, 20 studies were connected, among which, six were about lung cancer, four were about colorectal cancer, two were about prostate cancer, and another eight were about head and neck cancer, astrocytoma, oral cancer, ovarian cancer, breast cancer, gallbladder cancer, papillary thyroid carcinoma and hepatocellular carcinoma, respectively. Fifteen studies were Asian population based and five were Caucasion based. For *rs35285779* polymorphism, two studies were about lung cancer, another two were about prostate cancer and astrocytoma, respectively. All the four studies were Asian population based. For *rs35415678* polymorphism, two studies were connected to lung cancer and another two were about prostate cancer and astrocytoma, respectively. For *rs3923086* polymorphism, five studies were involved, two of which were about breast cancer and another three were oral cancer, papillary thyroid carcinoma and colorectal cancer, respectively. For *rs3923087* polymorphism, five studies were involved, two of which were about breast cancer and another three were oral cancer, ovarian cancer and colorectal cancer, respectively. For *rs4072245* polymorphism, there studies were about lung cancer, prostate cancer and astrocytoma, respectively. For *rs4791171* polymorphism, five studies were involved, two of which were about breast cancer and another three were colorectal cancer, oral cancer and gallbladder cancer, respectively. As to *rs7219582* polymorphism, four studies were included, two of which were about lung cancer, and another two were prostate cancer and astrocytoma, respectively. For *rs7224837* polymorphism, three studies were about oral cancer, ovarian cancer and bladder cancer, respectively. As to *rs9915936* polymorphism, four studies were included, two of which were focused on lung cancer, and another two were about prostate cancer and astrocytoma, respectively.

**Fig. 2:**
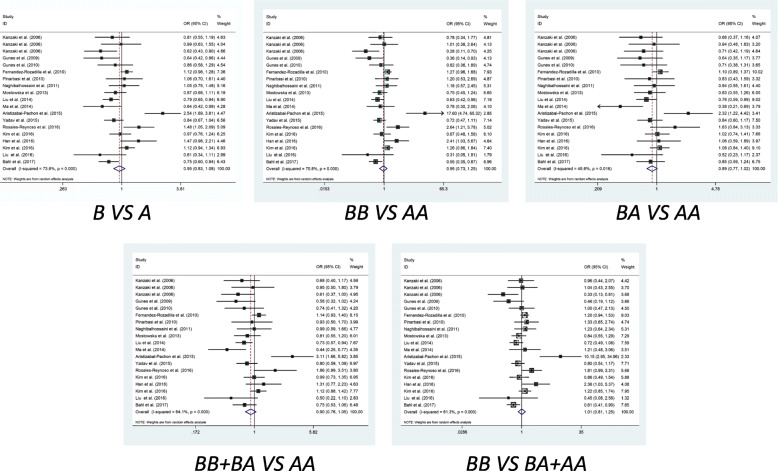
Correlation between *AXIN2 rs2240308* polymorphism and cancer susceptibility in five genetic models

Table 2 and Table S2 showed the results about *AXIN-2* polymorphisms and cancer susceptibility. There were significant associations in four genetic models between *rs11079571* polymorphism and overall cancer risk, including allelic contrast model (B vs. A: OR = 0.539, 95%CI = 0.478–0.609, PAdjust = 0.025), homozygote comparison model (BB vs. AA: OR = 0.22, 95% CI = 0.164–0.295, PAdjust< 0.001), heterozygote comparison model (BA vs. AA: OR = 0.292, 95% CI = 0.216–0.394, PAdjust< 0.001) and dominant comparison model (BB + BA vs. AA: OR = 0.249, 95% CI = 0.189–0.33, PAdjust< 0.001), whereas, there was no significant association in recessive comparison model (BB vs. BA+AA: OR = 0.619, 95% CI = 0.531–0.723, PAdjust = 0.11). What’s more, the stratification analysis of ethnicity also reflected *rs11079571* polymorphism risk to cancers in Asian population in B vs. A, BB vs. AA, BA vs. AA and BB + BA vs. AA models (PAdjust< 0.05). For cancer type analysis, *rs11079571* polymorphism showed strong association with risk of breast cancer in BA vs. AA and BB + BA vs. AA models (PAdjust< 0.05) (Table 2, Figure S1). For *rs1133683*, which had significant association with overall cancer risk in BB vs. AA and BB vs. BA+AA models (PAdjust< 0.05), and with Asian population in BB vs. BA+AA model (PAdjust< 0.05), with population based (PB) source of control in BB vs. AA and BB vs. BA+AA models (PAdjust< 0.05) (Table 2, Figure S2). For *rs2240308*, which showed significant correlation with risk of Asian population in BA vs. AA and BB + BA vs. AA models (PAdjust< 0.05) (Table 2, Fig. 2). For *rs35285779*, it was revealed significant association with overall cancer risk in BA vs. AA and BB + BA vs. AA models (PAdjust< 0.05) (Table 2, Figure S4). For *rs7219582*, it showed significant relationship with lung cancer risk in BA vs. AA and BB + BA vs. AA models (PAdjust< 0.05) (Table 2, Figure S10). For *rs9915936*, which also informed significant association with risk of PB source and lung cancer in BA vs. AA model (PAdjust< 0.05), respectively (Table 2, Figure S12). As to *rs2240307*, *rs35415678*, *rs3923086*, *rs3923087*, *rs4072245*, *rs4791171* and *rs7224837* polymorphisms, the pooled analysis data didn’t show any correlation with cancers, not only in overall risk, but also in cancer type, ethnicity or source of control (Table S2, Figure S3, S5, S6, S7, S8, S9, S11).

**Table 2:** Results of pooled analysis for *AXIN2* Polymorphism and cancer susceptibility

Polymorphism	Comparison	Subgroup	N	P_**H**_	P_**Z**_	P_**Adjust**_	OR & 95%CI (Random)	OR & 95%CI (Fixed)
rs11079571	B vs. A	Overall	3	< 0.001	0.005	0.025*	0.459(0.266–0.794)	0.539(0.478–0.609)
rs11079571	BB vs. AA	Overall	3	0.001	< 0.001	< 0.001*	0.2(0.085–0.469)	0.22(0.164–0.295)
rs11079571	BA vs. AA	Overall	3	0.081	< 0.001	< 0.001*	0.322(0.192–0.54)	0.292(0.216–0.394)
rs11079571	BB + BA vs. AA	Overall	3	0.08	< 0.001	< 0.001*	0.265(0.162–0.433)	0.249(0.189–0.33)
rs11079571	BB vs. BA+ AA	Overall	3	< 0.001	0.022	0.11	0.436(0.215–0.887)	0.619(0.531–0.723)
rs11079571	B vs. A	Asian	2	0.002	0.001	0.005*	0.351(0.191–0.646)	0.407(0.345–0.479)
rs11079571	BB vs. AA	Asian	2	0.007	< 0.001	< 0.001*	0.135(0.045–0.407)	0.178(0.127–0.251)
rs11079571	BA vs. AA	Asian	2	0.416	< 0.001	< 0.001*	0.246(0.174–0.346)	0.247(0.175–0.348)
rs11079571	BB + BA vs. AA	Asian	2	0.575	< 0.001	< 0.001*	0.216(0.157–0.297)	0.215(0.156–0.295)
rs11079571	BB vs. BA+ AA	Asian	2	< 0.001	0.083	0.415	0.311(0.083–1.166)	0.463(0.368–0.582)
rs11079571	B vs. A	Breast Cancer	2	< 0.001	0.148	0.74	0.446 (0.150–1.332)	0.594(0.507–0.697)
rs11079571	BB vs. AA	Breast Cancer	2	< 0.001	0.056	0.28	0.182(0.032–1.047)	0.209(0.133–0.329)
rs11079571	BA vs. AA	Breast Cancer	2	0.289	< 0.001	< 0.001*	0.419(0.255–0.689)	0.416(0.261–0.662)
rs11079571	BB + BA vs. AA	Breast Cancer	2	0.041	0.009	0.045*	0.294(0.118–0.734)	0.285(0.184–0.441)
rs11079571	BB vs. BA+ AA	Breast Cancer	2	< 0.001	0.202	1	0.356(0.073–1.74)	0.63(0.52–0.764)
rs1133683	B vs. A	Overall	6	< 0.001	0.664	1.000	1.076(0.773–1.498)	1.14(1.021–1.273)
rs1133683	BB vs. AA	Overall	6	< 0.001	0.005	0.025*	0.258(0.101–0.657)	0.391(0.284–0.539)
rs1133683	BA vs. AA	Overall	6	< 0.001	0.036	0.18	2.079(1.048–4.126)	2.298(1.948–2.71)
rs1133683	BB + BA vs. AA	Overall	6	< 0.001	0.1	0.5	1.78(0.895–3.538)	1.962(1.673–2.301)
rs1133683	BB vs. BA+ AA	Overall	6	< 0.001	< 0.001	< 0.001*	0.162(0.08–0.328)	0.206(0.152–0.278)
rs1133683	B vs. A	Asian	5	< 0.001	0.2	1.000	1.212(0.904–1.625)	1.25(1.11–1.408)
rs1133683	BB vs. AA	Asian	5	< 0.001	0.026	0.13	0.283(0.093–0.858)	0.469(0.329–0.67)
rs1133683	BA vs. AA	Asian	5	< 0.001	0.01	0.05	2.51(1.247–5.052)	2.627(2.203–3.132)
rs1133683	BB + BA vs. AA	Asian	5	< 0.001	0.025	0.125	2.186(1.105–4.322)	2.283(1.926–2.707)
rs1133683	BB vs. BA+ AA	Asian	5	< 0.001	< 0.001	< 0.001*	0.154(0.062–0.383)	0.21(0.15–0.295)
rs1133683	B vs. A	PB	4	< 0.001	0.828	1.000	1.051(0.67–1.651)	1.146(1.01–1.302)
rs1133683	BB vs. AA	PB	4	0.006	0.001	0.005*	0.256(0.113–0.584)	0.349(0.241–0.504)
rs1133683	BA vs. AA	PB	4	< 0.001	0.118	0.59	2.112(0.827–5.395)	2.541(2.093–3.084)
rs1133683	BB + BA vs. AA	PB	4	< 0.001	0.23	1.000	1.773(0.696–4.515)	2.142(1.777–2.582)
rs1133683	BB vs. BA+ AA	PB	4	0.045	< 0.001	< 0.001*	0.16(0.086–0.297)	0.184(0.13–0.259)
rs1133683	B vs. A	HB	2	0.004	0.72	1.000	1.127(0.587–2.163)	1.12(0.895–1.401)
rs1133683	BB vs. AA	HB	2	< 0.001	0.46	1.000	0.265(0.008–8.979)	0.556(0.291–1.062)
rs1133683	BA vs. AA	HB	2	< 0.001	0.249	1.000	2.001(0.615–6.508)	1.788(1.305–2.45)
rs1133683	BB + BA vs. AA	HB	2	< 0.001	0.361	1.000	1.782(0.515–6.161)	1.572(1.159–2.132)
rs1133683	BB vs. BA+ AA	HB	2	< 0.001	0.186	0.93	0.166(0.012–2.381)	0.297(0.158–0.559)
rs1133683	B vs. A	Lung Cancer	2	0.016	0.767	1.000	1.071(0.68–1.687)	1.196(1.023–1.399)
rs1133683	BB vs. AA	Lung Cancer	2	0.228	0.008	0.04*	0.491(0.263–0.918)	0.53(0.333–0.845)
rs1133683	BA vs. AA	Lung Cancer	2	< 0.001	0.317	1.000	2.143(0.482–9.522)	2.695(2.109–3.442)
rs1133683	BB + BA vs. AA	Lung Cancer	2	< 0.001	0.387	1.000	1.888(0.448–7.959)	2.36(1.86–2.995)
rs1133683	BB vs. BA+ AA	Lung Cancer	2	0.39	< 0.001	< 0.001*	0.21(0.136–0.325)	0.212(0.138–0.328)
rs1133683	B vs. A	Y	5	< 0.001	0.898	1.000	1.028(0.673–1.571)	1.036(0.899–1.193)
rs1133683	BB vs. AA	Y	5	< 0.001	0.008	0.04*	0.211(0.067–0.666)	0.293(0.195–0.44)
rs1133683	BA vs. AA	Y	5	< 0.001	0.14	0.7	1.767(0.83–3.762)	1.753(1.434–2.142)
rs1133683	BB + BA vs. AA	Y	5	< 0.001	0.287	1.000	1.512(0.706–3.241)	1.499(1.236–1.818)
rs1133683	BB vs. BA+ AA	Y	5	< 0.001	< 0.001	< 0.001*	0.152(0.057–0.405)	0.215(0.146–0.315)
rs2240308	B vs. A	Overall	20	< 0.001	0.402	1.000	0.949(0.841–1.072)	0.962(0.906–1.02)
rs2240308	BB vs. AA	Overall	19	< 0.001	0.722	1.000	0.952(0.726–1.248)	0.966(0.849–1.1)
rs2240308	BA vs. AA	Overall	19	0.016	0.089	0.445	0.887(0.773–1.018)	0.915(0.834–1.004)
rs2240308	BB + BA vs. AA	Overall	19	< 0.001	0.176	0.88	0.895(0.763–1.051)	0.923(0.846–1.007)
rs2240308	BB vs. BA+ AA	Overall	19	< 0.001	0.963	1.000	1.005(0.811–1.246)	1.006(0.895–1.13)
rs2240308	B vs. A	Asian	15	0.01	0.017	0.085	0.867(0.772–0.974)	0.879(0.815–0.947)
rs2240308	BB vs. AA	Asian	15	0.019	0.072	0.36	0.799(0.626–1.021)	0.806(0.686–0.946)
rs2240308	BA vs. AA	Asian	15	0.268	0.002	0.01*	0.828(0.731–0.939)	0.84(0.753–0.937)
rs2240308	BB + BA vs. AA	Asian	15	0.066	0.004	0.02*	0.811(0.704–0.934)	0.835(0.754–0.926)
rs2240308	BB vs. BA+ AA	Asian	15	0.053	0.273	1.000	0.889(0.721–1.097)	0.874(0.754–1.013)
rs2240308	B vs. A	Caucasian	5	< 0.001	0.138	0.69	1.228(0.936–1.61)	1.119(1.016–1.233)
rs2240308	BB vs. AA	Caucasian	4	< 0.001	0.082	0.41	2.069(0.912–4.692)	1.375(1.101–1.716)
rs2240308	BA vs. AA	Caucasian	4	0.044	0.224	1.000	1.253(0.871–1.801)	1.141(0.957–1.36)
rs2240308	BB + BA vs. AA	Caucasian	4	0.002	0.143	0.715	1.421(0.888–2.274)	1.198(1.014–1.414)
rs2240308	BB vs. BA+ AA	Caucasian	4	0.001	0.112	0.56	1.6(0.896–2.858)	1.277(1.053–1.548)
rs2240308	B vs. A	PB	12	< 0.001	0.894	1.000	0.987(0.815–1.195)	0.944(0.871–1.022)
rs2240308	BB vs. AA	PB	12	< 0.001	0.955	1.000	1.012(0.67–1.529)	0.919(0.777–1.087)
rs2240308	BA vs. AA	PB	12	0.064	0.364	1.000	0.924(0.78–1.096)	0.913(0.81–1.028)
rs2240308	BB + BA vs. AA	PB	12	< 0.001	0.644	1.000	0.949(0.762–1.183)	0.911(0.814–1.019)
rs2240308	BB vs. BA+ AA	PB	12	< 0.001	0.893	1.000	1.023(0.734–1.425)	0.96(0.823–1.119)
rs2240308	B vs. A	HB	8	0.114	0.719	1.000	0.935(0.822–1.064)	0.984(0.901–1.075)
rs2240308	BB vs. AA	HB	7	0.376	0.705	1.000	1.015(0.812–1.27)	1.04(0.849–1.273)
rs2240308	BA vs. AA	HB	7	0.047	0.1	0.5	0.839(0.681–1.034)	0.919(0.808–1.045)
rs2240308	BB + BA vs. AA	HB	7	0.043	0.128	0.64	0.855(0.699–1.046)	0.937(0.828–1.06)
rs2240308	BB vs. BA+ AA	HB	7	0.684	0.444	1.000	1.075(0.897–1.287)	1.073(0.896–1.284)
rs2240308	B vs. A	Colorectal Cancer	4	0.15	0.056	0.28	1.108(0.918–1.336)	1.116(0.997–1.249)
rs2240308	BB vs. AA	Colorectal Cancer	4	0.192	0.031	0.155	1.314(0.903–1.911)	1.295(1.024–1.637)
rs2240308	BA vs. AA	Colorectal Cancer	4	0.2	0.548	1.000	1.031(0.779–1.363)	1.057(0.882–1.266)
rs2240308	BB + BA vs. AA	Colorectal Cancer	4	0.113	0.252	1.000	1.083(0.794–1.478)	1.105(0.931–1.312)
rs2240308	BB vs. BA+ AA	Colorectal Cancer	4	0.563	0.036	0.18	1.241(1.011–1.524)	1.245(1.015–1.527)
rs2240308	B vs. A	Prostate Cancer	2	0.099	0.452	1.000	0.828(0.507–1.353)	0.832(0.619–1.119)
rs2240308	BB vs. AA	Prostate Cancer	2	0.509	0.987	1.000	1.004(0.539–1.869)	1.005(0.54–1.871)
rs2240308	BA vs. AA	Prostate Cancer	2	0.088	0.127	0.635	0.555(0.26–1.183)	0.542(0.35–0.84)
rs2240308	BB + BA vs. AA	Prostate Cancer	2	0.078	0.219	1.000	0.633(0.305–1.313)	0.62(0.412–0.934)
rs2240308	BB vs. BA+ AA	Prostate Cancer	2	0.872	0.39	1.000	1.284(0.726–2.27)	1.284(0.726–2.27)
rs2240308	B vs. A	Lung Cancer	6	< 0.001	0.176	0.88	0.854(0.678–1.074)	0.875(0.791–0.967)
rs2240308	BB vs. AA	Lung Cancer	6	< 0.001	0.199	0.995	0.714(0.427–1.194)	0.776(0.626–0.962)
rs2240308	BA vs. AA	Lung Cancer	6	0.317	0.069	0.345	0.868(0.736–1.023)	0.873(0.755–1.01)
rs2240308	BB + BA vs. AA	Lung Cancer	6	0.022	0.218	1.000	0.827(0.648–1.056)	0.857(0.747–0.983)
rs2240308	BB vs. BA+ AA	Lung Cancer	6	0.002	0.272	1.000	0.784(0.508–1.211)	0.817(0.669–0.998)
rs2240308	B vs. A	Y	16	< 0.001	0.099	0.495	0.899(0.792–1.02)	0.928(0.866–0.994)
rs2240308	BB vs. AA	Y	16	0.001	0.281	1.000	0.862(0.659–1.129)	0.904(0.78–1.048)
rs2240308	BA vs. AA	Y	16	0.097	0.018	0.09	0.843(0.732–0.972)	0.874(0.786–0.971)
rs2240308	BB + BA vs. AA	Y	16	0.008	0.03	0.15	0.838(0.714–0.983)	0.875(0.792–0.967)
rs2240308	BB vs. BA+ AA	Y	16	0.011	0.604	1.000	0.945(0.761–1.172)	0.962(0.843–1.099)
rs2240308	B vs. A	N	4	< 0.001	0.347	1.000	1.174(0.84–1.64)	1.056(0.944–1.182)
rs2240308	BB vs. AA	N	3	< 0.001	0.21	1.000	1.961(0.684–5.618)	1.199(0.922–1.561)
rs2240308	BA vs. AA	N	3	0.022	0.468	1.000	1.171(0.765–1.793)	1.066(0.88–1.292)
rs2240308	BB + BA vs. AA	N	3	0.001	0.347	1.000	1.304(0.75–2.265)	1.095(0.915–1.31)
rs2240308	BB vs. BA+ AA	N	3	< 0.001	0.243	1.000	1.648(0.713–3.81)	1.176(0.919–1.504)
rs35285779	B vs. A	Overall	4	0.068	0.011	0.055	0.603(0.409–0.889)	0.632(0.496–0.806)
rs35285779	BB vs. AA	Overall	4	0.378	0.038	0.19	0.43(0.194–0.955)	0.368(0.176–0.77)
rs35285779	BA vs. AA	Overall	4	0.384	0.009	0.045*	0.685(0.513–0.915)	0.684(0.514–0.909)
rs35285779	BB + BA vs. AA	Overall	4	0.155	0.001	0.005*	0.613(0.421–0.893)	0.639(0.486–0.839)
rs35285779	BB vs. BA+ AA	Overall	4	0.448	0.017	0.085	0.473(0.219–1.025)	0.408(0.195–0.853)
rs35285779	B vs. A	PB	2	0.172	0.034	0.17	0.691(0.442–1.08)	0.711(0.519–0.975)
rs35285779	BB vs. AA	PB	2	0.352	0.145	0.725	0.452(0.147–1.388)	0.443(0.148–1.323)
rs35285779	BA vs. AA	PB	2	0.434	0.102	0.51	0.741(0.517–1.062)	0.741(0.517–1.062)
rs35285779	BB + BA vs. AA	PB	2	0.257	0.057	0.285	0.702(0.467–1.054)	0.713(0.504–1.01)
rs35285779	BB vs. BA+ AA	PB	2	0.393	0.197	0.985	0.498(0.163–1.52)	0.488(0.164–1.452)
rs35285779	B vs. A	HB	2	0.041	0.103	0.515	0.508(0.226–1.145)	0.535(0.365–0.783)
rs35285779	BB vs. AA	HB	2	0.131	0.025	0.125	0.262(0.028–2.443)	0.317(0.116–0.868)
rs35285779	BA vs. AA	HB	2	0.161	0.031	0.155	0.592(0.305–1.149)	0.598(0.375–0.954)
rs35285779	BB + BA vs. AA	HB	2	0.081	0.104	0.52	0.519(0.236–1.144)	0.535(0.344–0.833)
rs35285779	BB vs. BA+ AA	HB	2	0.16	0.043	0.215	0.311(0.041–2.355)	0.354(0.13–0.967)
rs35285779	B vs. A	Lung Cancer	2	0.172	0.034	0.17	0.691(0.442–1.08)	0.711(0.519–0.975)
rs35285779	BB vs. AA	Lung Cancer	2	0.352	0.145	0.725	0.452(0.147–1.388)	0.443(0.148–1.323)
rs35285779	BA vs. AA	Lung Cancer	2	0.434	0.102	0.51	0.741(0.517–1.062)	0.741(0.517–1.062)
rs35285779	BB + BA vs. AA	Lung Cancer	2	0.257	0.057	0.285	0.702(0.467–1.054)	0.713(0.504–1.01)
rs35285779	BB vs. BA+ AA	Lung Cancer	2	0.393	0.197	0.985	0.498(0.163–1.52)	0.488(0.164–1.452)
rs7219582	B vs. A	Overall	4	0.386	0.077	0.385	0.822(0.645–1.048)	0.82(0.659–1.021)
rs7219582	BA vs. AA	Overall	4	0.035	0.538	1.000	0.75(0.3–1.873)	0.491(0.344–0.7)
rs7219582	BB + BA vs. AA	Overall	4	0.045	0.538	1.000	0.758(0.313–1.833)	0.51(0.358–0.727)
rs7219582	B vs. A	Lung Cancer	2	0.941	0.041	0.205	0.789(0.629–0.99)	0.789(0.629–0.99)
rs7219582	BA vs. AA	Lung Cancer	2	0.399	< 0.001	< 0.001*	0.394(0.265–0.586)	0.394(0.265–0.585)
rs7219582	BB + BA vs. AA	Lung Cancer	2	0.436	< 0.001	< 0.001*	0.414(0.278–0.614)	0.413(0.278–0.614)
rs9915936	B vs. A	Overall	4	0.873	0.038	0.19	0.708(0.51–0.981)	0.707(0.51–0.981)
rs9915936	BA vs. AA	Overall	4	0.668	0.014	0.07	0.634(0.44–0.914)	0.633(0.44–0.91)
rs9915936	BB + BA vs. AA	Overall	4	0.775	0.021	0.105	0.662(0.466–0.94)	0.661(0.466–0.939)
rs9915936	B vs. A	PB	2	0.806	0.034	0.17	0.667(0.459–0.971)	0.667(0.459–0.97)
rs9915936	BA vs. AA	PB	2	0.548	0.009	0.045*	0.567(0.369–0.871)	0.566(0.369–0.87)
rs9915936	BB + BA vs. AA	PB	2	0.669	0.016	0.08	0.607(0.404–0.913)	0.607(0.404–0.912)
rs9915936	B vs. A	HB	2	0.619	0.646	1.000	0.855(0.436–1.677)	0.854(0.436–1.674)
rs9915936	BA vs. AA	HB	2	0.608	0.637	1.000	0.848(0.425–1.692)	0.847(0.425–1.689)
rs9915936	BB + BA vs. AA	HB	2	0.608	0.637	1.000	0.848(0.425–1.692)	0.847(0.425–1.689)
rs9915936	B vs. A	Lung Cancer	2	0.806	0.034	0.17	0.667(0.459–0.971)	0.667(0.459–0.97)
rs9915936	BA vs. AA	Lung Cancer	2	0.548	0.009	0.045*	0.567(0.369–0.871)	0.566(0.369–0.87)
rs9915936	BB + BA vs. AA	Lung Cancer	2	0.669	0.016	0.08	0.607(0.404–0.913)	0.607(0.404–0.912)

*P*_***H***_ *P* value of Q test for heterogeneity test, *P*_*Z*_ *P* value of meta-analysis, *P*_*Adjust*_ Adjust *P*_*Z*_ value by Bonferroni corrections, *P*_*Adjust*_ = *P*_*Z*_ * 5, *P-B* Population based, *HWE* Hardy Weinberg Equilibrium, *Y* polymorphisms conformed to HWE in the control group, *N* polymorphisms didn’t conform to HWE in the control group

* *P* value less than 0.05 was considered as statistically significant

### Sensitivity analysis and publication bias

**Fig. 3:**
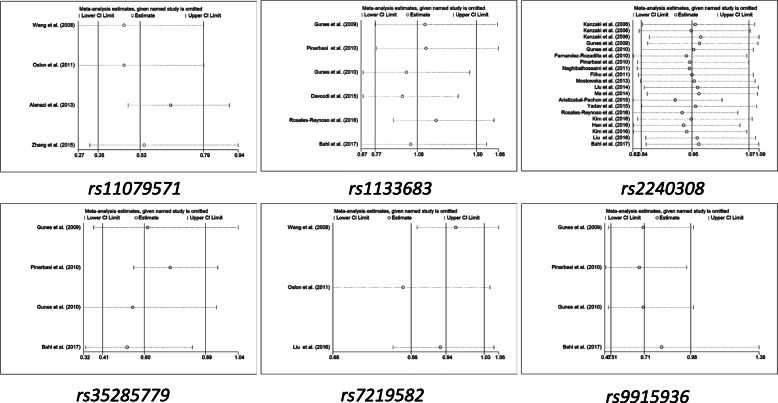
Sensitivity analysis of *AXIN2* polymorphisms and overall cancers **(B vs. A).** The results of *rs11079571*, *rs1133683*, *rs2240308*, *rs35285779*, *rs7219582*, *rs9915936* were presented in this figure. The dotted area represents the 95% confidence interval

**Fig. 4:**
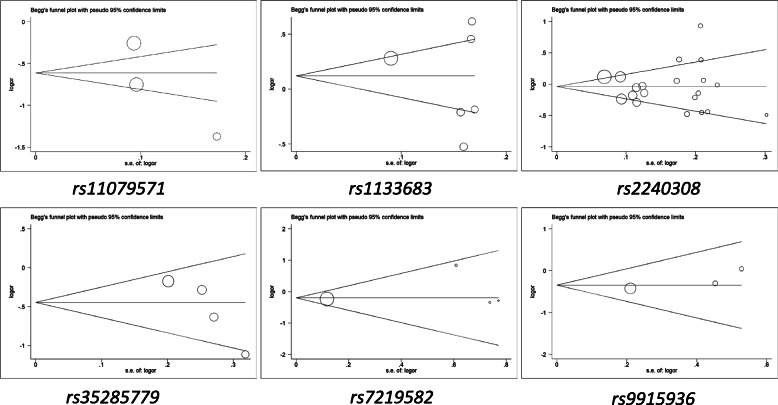
Begg’s plot for publication bias of *AXIN2* polymorphisms and overall cancers **(B vs. A).** The results of *rs11079571*, *rs1133683*, *rs2240308*, *rs35285779*, *rs7219582*, *rs9915936* were presented in this figure. The x-axis stands for the value of log (OR), and the y-axis stands for the value of natural logarithm of OR. The horizontal line stands for the overall estimated value of log (OR). The two diagonal lines in the figure represent the pseudo 95% confidence limits of the effect estimate

To check the influence of individual study on overall data, we applied sensitivity analysis, and the results of the pooled analysis proved that the OR value was not influenced by individual study (Fig. 3, S13 and Table S3). At the same time, to evaluate the publication bias, Begg’s funnel plot and Egger’s test were performed, and the results didn’t show asymmetric evidence (Fig. 4, S14 and Table S4).

### Linkage disequilibrium (LD) analysis of *AXIN-2* polymorphisms

**Fig. 5:**
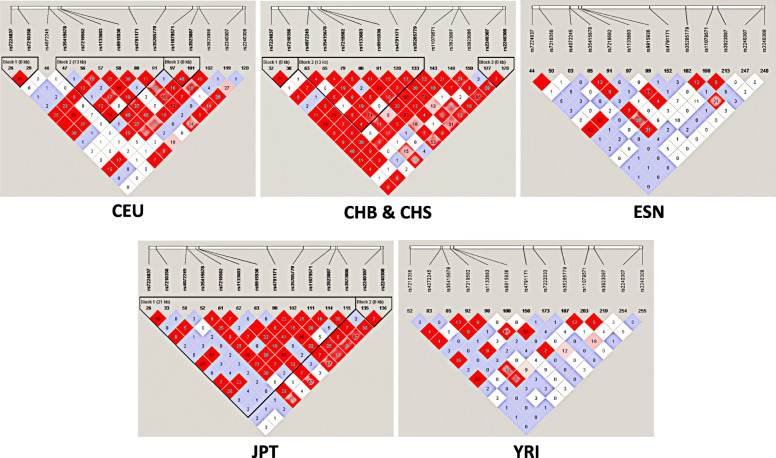
LD analysis for *AXIN-2* polymorphisms in different populations acquired from 1000 Genomes Project. The value of r^2^ is showed in each square, and white colors represent no significant LD between different polymorphisms. CEU: Utah residents with Northern and Western European ancestry from the CEPH collection; CHB: Han Chinese in Beijing, China; CHS: Southern Han Chinese, China; ESN: Esan in Nigeria; JPT: Japanese in Tokyo, Japan; YRI: Yoruba in Ibadan, Nigeria

LD analysis was assessed to evaluate the inner interaction of each *AXIN-2* polymorphism and the results were shown in Fig. 5. Obviously, there was significant LD between *rs7224837* and *rs7210356* in CEU populations (*r*^2^ = 0.91), the same as between *rs7210356* and *rs9915936* (*r*^2^ = 0.91), *rs1133683* and *rs4791171* (*r*^2^ = 0.85), *rs35415678* and *rs35285779* (*r*^2^ = 0.84). There was significant LD between *rs7224837* and *rs9915936* in CHB&CHS populations (*r*^2^ = 0.93), the same as between *rs1133683* and *rs4791171* (*r*^2^ = 0.93), *rs1133683* and *rs3923087* (*r*^2^ = 0.83). There was significant LD between *rs7224837* and *rs9915936* in ESN populations (*r*^2^ = 0.62), the same as between *rs7210356* and *rs9915936* (*r*^2^ = 0.62), *rs1133683* and *rs4791171* (*r*^2^ = 0.66). There was significant LD between *rs7224837* and *rs9915936* in JPT populations (*r*^2^ = 0.95), the same as between *rs35415678* and *rs35285779* (*r*^2^ = 0.90), *rs1133683* and *rs3923087* (*r*^2^ = 0.95), *rs4791171* and *rs3923087* (*r*^2^ = 0.95). There was significant LD between *rs7210356* and *rs7222033* in YRI populations (*r*^2^ = 0.67), the same as between *rs9915936* and *rs7222033* (*r*^2^ = 0.54).

## Discussion

*AXIN2* plays an important role as a negative regulator in regulating β-catenin stability. As β-catenin was well studied as an important gene related to many cancers [47–50], the correlation between *AXIN2* and tumor progression and metastasis have also been well reported by many studies in the past few decades. Xie et al. [51] reported *AXIN2* can be targeted by miR143HG/miR-1275 to regulate breast cancer progression by modulating the Wnt/β-catenin pathway. Ren et al. [52] revealed that *AXIN2* was a target of miR-454-3p and was involved in the activation of Wnt/β-catenin signaling, which can be suppressed by miR-454-3p to promote metastasis and the stemness of breast cancer. Chen et al. [11] demonstrated that *AXIN2* could be down-regulated by miR-544, thus to promote human osteosarcoma cell proliferation. Lu et al. [53] reported that *AXIN2* was identified to be a functional downstream target of miR-374a, and decreased expression of Axin2 could promote OS cell proliferation.

Previous studies have also demonstrated the association between *AXIN2* and cancer risk and susceptibility. Liu et al. [15] reported that *AXIN2 rs11655966* and *rs3923086* polymorphism had significant associations with papillary thyroid carcinoma. Aristizabal-Pachon et al. [42] showed significant association between *AXIN2 rs151279728* and *rs2240308* polymorphisms and breast cancer susceptibility. Ma et al. [40] concluded that there was a significant correlation between *rs2240308* polymorphism and the susceptibility of prostate cancer, while E·Pinarbasi et al. [16] reported that there was no significant correlation between prostate cancer susceptibility and *rs2240308* polymorphism in Turkish population.

Judge from the studies related to *AXIN2* polymorphism and cancer risk and susceptibility, the results seem not consistent. So, we preformed this meta-analysis to the current evidence for *AXIN2* polymorphism to cancer risk. As the results showed in Figures and Tables, we concluded that *AXIN2 rs11079571* had significant correlation with overall cancers and Asian population subtype. As for other polymorphisms, like *rs1133683* and *rs35285779* had significant correction with overall cancers in two genetic models (*rs1133683,* BB vs. AA and BB vs. BA+ AA) (*rs35285779*, BA vs. AA and BB + BA vs. AA), however, the others had no strong relationship with overall cancer risk. As to subtype cancers, *rs11079571* showed significant correlation with breast cancer, *rs1133683*, *rs7219582* and *rs9915936* indicated significant correlation with lung cancer. What’s more, the LD analysis showed a significant LD between *rs7224837* and *rs7210356/rs9915936*, as well as between *rs9915936* and *rs7210356/rs7224837*, which means that maybe we should combine two or more polymorphisms to analysis the correlation between *AXIN2* and cancer risk and susceptibility in future.

At the same time, we must realize the limitations that exist in this study. Firstly, an enlarged numbers of articles that involved are needed in the analysis, especially for *AXIN2 rs7224837* polymorphism. Secondly, when we searched the articles, we only involved the studies in English and Chinese, which may also cause bias for not involving other languages. Thirdly, for subtype analysis, we didn’t analyze every cancer for each polymorphism, which may lead to some shortcomings. Fourthly, gene-environment interactions were ignored in this study because of lack necessary data.

## Conclusions

In conclusion, our updated study suggests that *AXIN2 rs11079571, rs1133683* and *rs35285779* polymorphisms are associated with overall cancer susceptibility, which may provide a new insight to understand the correlation between AXIN2 gene and cancer risk. What’s more, the combination of two or more polymorphisms may benefit us to better understand the function of AXIN2 polymorphisms in different populations. Future large scale and well-designed research are required to validate these effects in more detail.

## Supplementary Information

**Additional file 1 **: **Table S1.** Methodological quality of the included studies according to the Newcastle-Ottawa Scale. **Table S2.** Results of pooled analysis for AXIN2 Polymorphism and cancer susceptibility. **Table S3.** Details of the sensitivity analyses for AXIN2 polymorphism and urinary cancer risk. **Table S4.** *P* values of the Egger’s test for AXIN2 polymorphism.

**Additional file 2 **: **Figure S1.** Meta-analysis ofAXIN2-rs11079571 polymorphism and overall cancer risk in 5 genetic models.

**Additional file 3 **: **Figure S2.** Meta-analysis ofAXIN2-rs1133683 polymorphism and overall cancer risk in 5 genetic models.

**Additional file 4 **: **Figure S3.** Meta-analysis ofAXIN2-rs2240307 polymorphism and overall cancer risk in 3 genetic models.

**Additional file 5 **: **Figure S4.** Meta-analysis ofAXIN2-rs35285779 polymorphism and overall cancer risk in 5 genetic models.

**Additional file 6 **: **Figure S5.** Meta-analysis ofAXIN2-rs35415678 polymorphism and overall cancer risk in 3 genetic models.

**Additional file 7 **: **Figure S6.** Meta-analysis ofAXIN2-rs3923086 polymorphism and overall cancer risk in 5 genetic models.

**Additional file 8 **: **Figure S7.** Meta-analysis ofAXIN2-rs3923087 polymorphism and overall cancer risk in 5 genetic models.

**Additional file 9 **: **Figure S8.** Meta-analysis ofAXIN2-rs4072245 polymorphism and overall cancer risk in 3 genetic models.

**Additional file 10 **: **Figure S9.** Meta-analysis ofAXIN2-rs4791171 polymorphism and overall cancer risk in 5 genetic models.

**Additional file 11 **: **Figure S10.** Meta-analysis ofAXIN2-rs7219582 polymorphism and overall cancer risk in 5 genetic models.

**Additional file 12 **: **Figure S11.** Meta-analysis ofAXIN2-rs7224837 polymorphism and overall cancer risk in 5 genetic models.

**Additional file 13 **: **Figure S12.** Meta-analysis ofAXIN2-rs9915936 polymorphism and overall cancer risk in 5 genetic models.

**Additional file 14 **: **Figure S13.** Sensitivity analysis ofAXIN2 polymorphism and overall cancer (Bvs.A). The results of *rs2240307*, *rs35415678*, *rs3923086*, *rs3923087*, *rs4072245*, *rs4791171, rs7210356, rs7224837* were presented in this figure. The dotted area represents the 95% confidence interval.

**Additional file 15 **: **Figure S14.** Begg’splot ofAXIN2 polymorphism and overall cancer (Bvs.A). The results of *rs2240307*, *rs35415678*, *rs3923086*, *rs3923087*, *rs4072245*, *rs4791171, rs7224837* were presented in this figure. The x-axis stands for the value of log (OR), and the y-axis stands for the value of natural logarithm of OR. The horizontal line stands for the overall estimated value of log (OR). The two diagonal lines in the figure represent the pseudo 95% confidence limits of the effect estimate.

## Data Availability

All data are presented in the figures, tables and supplementary files.

## Abbreviations

LD: Linkage disequilibrium
HWE: Hardy–Weinberg equilibrium
NOS: Newcastle-Ottawa Scale
PB: Population based
CHS: Southern Han Chinese, China
CHB: Han Chinese in Beijing, China
ESN: Esan in Nigeria
YRI: Yoruba in Ibadan, Nigeria
JPT: Japanese in Tokyo, Japan
CRC: Colorectal Cancer
PTC: Papillary Thyroid Carcinoma

## References

[1] Siegel RL, Miller KD, Jemal A (2019). Cancer statistics, 2019. CA Cancer J Clin.

[2] Chen W, Zheng R, Baade PD, Zhang S, Zeng H, Bray F, Jemal A, Yu XQ, He J (2016). Cancer statistics in China, 2015. CA Cancer J Clin.

[3] Ferlay J, Colombet M, Soerjomataram I, Dyba T, Randi G, Bettio M, Gavin A, Visser O, Bray F (2018). Cancer incidence and mortality patterns in Europe: estimates for 40 countries and 25 major cancers in 2018. Eur J Cancer.

[4] Hongdan L, Feng L (2018). miR-3120-5p promotes colon cancer stem cell stemness and invasiveness through targeting Axin2. Biochem Biophys Res Commun.

[5] Amalia R, Abdelaziz M, Puteri MU, Hwang J, Anwar F, Watanabe Y, Kato M (2019). TMEPAI/PMEPA1 inhibits Wnt signaling by regulating β-catenin stability and nuclear accumulation in triple negative breast cancer cells. Cell Signal.

[6] Zhang X, Kim K, Zheng Z (2017). Snail and Axin2 expression predict the malignant transformation of oral leukoplakia. Oral Oncol.

[7] Li Y, Jin K, van Pelt GW, van Dam H, Yu X, Mesker WE, ten Dijke P, Zhou F, Zhang L (2016). C-Myb enhances breast cancer invasion and metastasis through the Wnt/β-catenin/Axin2 pathway. Cancer Res.

[8] Dong X, Seelan RS, Qian C, Mai M, Liu W (2001). Genomic structure, chromosome mapping and expression analysis of the human AXIN2 gene. Cytogenet Genome Res.

[9] Mai M, Qian C, Yokomizo A, Smith DI, Liu W (1999). Cloning of the human homolog of conductin (AXIN2), a gene mapping to chromosome 17q23- q24. Genomics..

[10] Chen D, Li SG, Chen JY, Xiao M (2018). MiR-183 maintains canonical Wnt signaling activity and regulates growth and apoptosis in bladder cancer via targeting AXIN2. Eur Rev Med Pharmacol Sci.

[11] Chen M, Liu YY, Zheng MQ, Wang XL, Gao XH, Chen L, Zhang GM (2018). microRNA-544 promoted human osteosarcoma cell proliferation by downregulating AXIN2 expression. Oncol Lett.

[12] Kim WK, Byun WS, Chung H (2018). Esculetin suppresses tumor growth and metastasis by targeting Axin2/E-cadherin axis in colorectal cancer. Biochem Pharmacol.

[13] Otero L, Lacunza E, Vasquez V, Arbelaez V, Cardier F, González F (2019). Variations in AXIN2 predict risk and prognosis of colorectal cancer. BDJ Open.

[14] Zhong A, Pan X, Shi M, Xu H (2015). 148 C/T polymorphism of Axin2 contributes to a decreased risk of cancer: evidence from a meta-analysis. Onco Targets Ther..

[15] Liu X, Li S, Lin X, Yan K, Zhao L, Yu Q, Liu X (2016). AXIN2 is associated with papillary thyroid carcinoma. Iran Red Crescent Med J.

[16] Pinarbasi E, Gunes EG, Pinarbasi H, Donmez G, Silig Y (2011). AXIN2 polymorphism and its association with prostate cancer in a Turkish population. Med Oncol.

[17] Xu B, Yuan W, Shi L, Zuo L, Wu XY, Zhang W, Wen Q (2019). New insights into the association between AXIN2 148 C/T, 1365 C/T, and rs4791171 A/G variants and cancer risk. Cancer Cell Int.

[18] Dai F, Zhu L, Zhang W (2019). The association between three AXIN2 variants and cancer risk. J Cell Biochem.

[19] Bonferroni CE (1936). Teoria Statistica Delle Classi e Calcolo Delle Probabilità. Comm Firenze.

[20] DerSimonian R, Laird N (1986). Meta-analysis in clinical trials. Control Clin Trials.

[21] Yu Y, Tao Y, Liu L, Yang J, Wang L, Li X, Zhuang X, Chu M (2017). New concept of the Axin2 rs2240308 polymorphism and cancer risk: an updated meta-analysis. Neoplasma..

[22] Thakkinstian A, McElduff P, D’Este C (2005). A method for meta-analysis of molecular association studies. Stat Med.

[23] Begg CB, Mazumdar M (1994). Operating characteristics of a rank correlation test for publication bias. Biometrics..

[24] Higgins JP, Thompson SG (2002). Quantifing heterogeneity in a meta-analysis. Stat Med.

[25] Wang X, Goode EL, Fredericksen ZS, Vierkant RA, Pankratz VS, Liu-Mares W, Rider DN, Vachon CM, Cerhan JR, Olson JE, Couch FJ (2008). Association of genetic variation in genes implicated in the β-catenin destruction complex with risk of breast cancer. Cancer Epidemiol Biomark Prev.

[26] Alanazi MS, Parine NR, Shaik JP, Alabdulkarim HA, Ajaj SA, Khan Z (2013). Association of single nucleotide polymorphisms in Wnt signaling pathway genes with breast cancer in Saudi patients. PLoS One.

[27] Zhang L, Zhou J, Ye Y (2015). Genetic polymorphisms in Wnt signaling pathway and acute leukemia. J Sichuan Univ (Med Sci Edi).

[28] Gunes EG, Pinarbasi E, Pinarbasi H, Silig Y (2009). Strong association between lung cancer and the AXIN2 polymorphism. Mol Med Rep.

[29] Bahl C, Sharma S, Singh N (2017). Association study between genetic variations in *Axin2* gene and lung cancer risk in north Indian population: a multiple interaction analysis. Tumor Biol.

[30] Gunes EG, Pinarbasi E, Pinarbasi H (2010). AXIN2 polymorphism and its association with astrocytoma in a Turkish population. Mol Med Rep.

[31] Mostowska A, Pawlik P, Sajdak S (2013). An analysis of polymorphisms within the Wnt signaling pathway in relation to ovarian cancer risk in a polish population. Mol Diagn Ther.

[32] Rosales-Reynoso MA, Arredondo-Valdez AR, Wence-Chávez LI, Barros-Núñez P, Gallegos-Arreola MP, Flores-Martínez SE, Sánchez-Corona J (2016). *AXIN2* polymorphisms and their association with colorectal cancer in Mexican patients. Genet Test Mol Biomarkers.

[33] Han S, Lv L, Wang X (2016). Association of AXIN2 and MMP7 polymorphisms with non-small cell lung cancer in Chinese Han population. Int J Clin Exp Pathol.

[34] Andrade Filho PA, Letra A, Cramer A, Prasad JL, Garlet GP, Vieira AR, Ferris RL, Menezes R (2011). Insights from studies with oral cleft genes suggest associations between WNT-pathway genes and risk of oral cancer. J Dent Res.

[35] Kanzaki H, Ouchida M, Hanafusa H, Yano M, Suzuki H, Aoe M, Imai K, Shimizu N, Nakachi K, Shimizu K (2006). Single nucleotide polymorphism of the AXIN2 gene is preferentially associated with human lung cancer risk in a Japanese population. Int J Mol Med.

[36] Liu D, Li L, Yang Y, Liu W, Wu J (2014). The Axin2 rs2240308 polymorphism and susceptibility to lung cancer in a Chinese population. Tumor Biol.

[37] Kim CH. Environmental tobacco smoke, genetic susceptibility, and lung cancer among never smokers: ULCA; 2016. ProQuest ID: Kim_UCLA_0031D_14561. Merritt ID: ark:/13030/m5xt0h1p. Retrieved from https://escholarship.org/uc/item/81j69493

[38] Fernández-Rozadilla C, de Castro L, Clofent J, Brea-Fernández A, Bessa X, Abulí A, Andreu M, Jover R, Xicola R, Llor X, Castells A, Castellví-Bel S, Carracedo A, Ruiz-Ponte C, for the Gastrointestinal Oncology Group of the Spanish Gastroenterological Association (2010). Single nucleotide polymorphisms in the Wnt and BMP pathways and colorectal cancer risk in a Spanish cohort. PLoS One.

[39] Naghibalhossaini F, Zamani M, Mokarram P, Khalili I, Rasti M, Mostafavi-pour Z (2012). Epigenetic and genetic analysis of WNT signaling pathway in sporadic colorectal cancer patients from Iran. Mol Biol Rep.

[40] Ma C, Liu C, Huang P (2014). Significant association between the Axin2 rs2240308 single nucleotide polymorphism and the incidence of prostate cancer. Oncol Lett.

[41] Mostowska A, Pawlik P, Sajdak S, Markowska J, Pawałowska M, Lianeri M, Jagodzinski PP (2014). An analysis of polymorphisms within the Wnt signaling pathway in relation to ovarian cancer risk in a polish population. Mol Diagn Ther..

[42] Aristizabal-Pachon AF, Carvalho TI, Carrara HH, Andrade J, Takahashi CS (2015). AXIN2 polymorphisms, the β-catenin destruction complex expression profile and breast cancer susceptibility. Asian Pac J Cancer Prev.

[43] Yadav A, Gupta A, Yadav S, Rastogi N, Agrawal S, Kumar A, Kumar V, Misra S, Mittal B (2016). Association of Wnt signaling pathway genetic variants in gallbladder cancer susceptibility and survival. Tumor Biol.

[44] Kim SS, Cho HJ, Lee H (2016). Genetic polymorphisms in the Wnt/β-catenin pathway genes as predictors of tumor development and survival in patients with hepatitis B virus-associated hepatocellular carcinoma. Clin Biochem.

[45] Parine NR, Azzam NA, Shaik J, Aljebreen AM, Alharbi O, Almadi MA, Alanazi M, Khan Z (2019). Genetic variants in the WNT signaling pathway are protectively associated with colorectal cancer in a Saudi population. Saudi J Biol Sci.

[46] Pierzynski JA, Hildebrandt MA, Kamat AM, Lin J, Ye Y, Dinney CPN, Wu X (2015). Genetic variants in the Wnt/β-catenin signaling pathway as indicators of bladder cancer risk. J Urol.

[47] Al-Dhfyan A, Alhoshani A, Korashy HM (2017). Aryl hydrocarbon receptor/cytochrome P450 1A1 pathway mediates breast cancer stem cells expansion through PTEN inhibition and β-catenin and Akt activation. Mol Cancer.

[48] Persad S, Troussard AA, McPhee TR (2001). Tumor suppressor PTEN inhibits nuclear accumulation of beta-catenin and T cell/lymphoid enhancer factor 1-mediated transcriptional activation. J Cell Biol.

[49] Ma J, Guo X, Zhang J, Wu D, Hu X, Li J, Lan Q, Liu Y, Dong W (2017). PTEN gene induces cell invasion and migration via regulating AKT/GSK-3β/β-catenin signaling pathway in human gastric cancer. Dig Dis Sci.

[50] Ge H, Liang C, Li Z, An D, Ren S, Yue C, Wu J (2018). DcR3 induces proliferation, migration, invasion, and EMT in gastric cancer cells via the PI3K/AKT/GSK-3β/β-catenin signaling pathway. Onco Targets Ther.

[51] Xie H, Huang H, Huang W (2018). LncRNA miR143HG suppresses bladder cancer development through inactivating Wnt/β-catenin pathway by modulating miR-1275/AXIN2 axis. J Cell Physiol.

[52] Ren L, Chen H, Song J, Chen X, Lin C, Zhang X, Hou N, Pan J, Zhou Z, Wang L, Huang D, Yang J, Liang Y, Li J, Huang H, Jiang L (2019). MiR-454-3p-mediated Wnt/β-catenin signaling antagonists suppression promotes breast cancer metastasis. Theranostics..

[53] Lu T, Zhang C, Chai MX, An YB, Jia JL (2015). MiR-374a promotes the proliferation of osteosarcoma cell proliferation by targeting Axin2. Int J Clin Exp Pathol.

