# Supplementary material for: New insights of the correlation between AXIN2 polymorphism and cancer risk and susceptibility: evidence from 72 studies
Source: BMC Cancer. 2021 Apr 1;21:353. doi: 10.1186/s12885-021-08092-0 (PMC8017882; doi:10.1186/s12885-021-08092-0)
Supplement: Supplementary file 1 — Additional file 1 : Table S1. Methodological quality of the included studies according to the Newcastle-Ottawa Scale. Table S2. Results of pooled analysis for AXIN2 Polymorphism and cancer susceptibility. Table S3. Details of the sensitivity analyses for AXIN2 polymorphism and urinary cancer risk. Table S4. P values of the Egger’s test for AXIN2 polymorphism. [file 12885_2021_8092_MOESM1_ESM.doc]

Supplementary table 1. Methodological quality of the included studies according to the Newcastle-Ottawa Scale.

| **SNP** | **Author** | **Year** | **Adequacy of Case Definition** | **Representativeness of the Cases** | **Selection of Controls** | **Definition of Controls** | **Comparability Cases/Controls** | **Ascertainment of Exposure** | **Same Method of Ascertainment** | **Non-response rate** |
| --- | --- | --- | --- | --- | --- | --- | --- | --- | --- | --- |
| rs11079571 | Wang et al. | 2008 | * | * | * | * | ** | * | * | NA |
| rs11079571 | Alanazi et al. | 2013 | * | * | * | * | ** | * | * | NA |
| rs11079571 | Zhang et al. | 2015 | * | * | * | * | * | * | * | NA |
| rs1133683 | Gunes et al. | 2009 | * | * | * | * |  | * | * | NA |
| rs1133683 | Pinarbasi et al. | 2010 | * | * |  |  | * | * | * | NA |
| rs1133683 | Gunes et al. | 2010 | * | * |  |  | ** | * | * | NA |
| rs1133683 | Davoodi et al. | 2015 | * |  | * |  |  |  | * | NA |
| rs1133683 | Rosales-Reynoso et al. | 2016 | * | * | * | * |  | * | * | NA |
| rs1133683 | Bahl et al. | 2017 | * | * | * | * | ** | * | * | NA |
| rs2240307 | Gunes et al. | 2009 | * | * | * | * |  | * | * | NA |
| rs2240307 | Pinarbasi et al. | 2010 | * | * |  |  | * | * | * | NA |
| rs2240307 | Gunes et al. | 2010 | * | * |  |  | ** | * | * | NA |
| rs2240307 | Filho et al. | 2011 | * | * |  |  | * | * | * | NA |
| rs2240307 | Han et al. | 2016 | * | * | * | * | * | * | * | NA |
| rs2240307 | Bahl et al. | 2017 | * | * | * | * | ** | * | * | NA |
| rs2240308 | Kanzaki et al. | 2006 | * | * | * | * | ** | * | * | NA |
| rs2240308 | Kanzaki et al. | 2006 | * | * | * | * | ** | * | * | NA |
| rs2240308 | Kanzaki et al. | 2006 | * | * | * | * | ** | * | * | NA |
| rs2240308 | Gunes et al. | 2009 | * | * | * | * |  | * | * | NA |
| rs2240308 | Gunes et al. | 2010 | * | * |  |  | ** | * | * | NA |
| rs2240308 | Ferna´ndez-Rozadilla et al. | 2010 | * | * |  |  | ** | * | * | NA |
| rs2240308 | Pinarbasi et al. | 2010 | * | * |  |  | * | * | * | NA |
| rs2240308 | Naghibalhossaini et al. | 2011 | * | * | * | * | * | * | * | NA |
| rs2240308 | Filho et al. | 2011 | * | * |  |  | * | * | * | NA |
| rs2240308 | Mostowska et al. | 2013 | * | * |  |  | * | * | * | NA |
| rs2240308 | Liu et al. | 2014 | * | * | * | * | ** | * | * | NA |
| rs2240308 | Ma et al. | 2014 | * | * |  |  | * | ** | * | NA |
| rs2240308 | Aristizabal-Pachon et al. | 2015 | * | * | * | * | ** | * | * | NA |
| rs2240308 | Yadav et al. | 2015 | * | * | * | * | ** | * | * | NA |
| rs2240308 | Rosales-Reynoso et al. | 2016 | * | * | * | * |  | * | * | NA |
| rs2240308 | Kim et al. | 2016 | * |  |  |  |  |  | * | NA |
| rs2240308 | Han et al. | 2016 | * | * | * | * | * | * | * | NA |
| rs2240308 | Kim et al. | 2016 | * |  | * |  |  |  | * | NA |
| rs2240308 | Liu et al. | 2016 | * | * |  |  | ** | * | * | NA |
| rs2240308 | Bahl et al. | 2017 | * | * | * | * | ** | * | * | NA |
| rs35285779 | Gunes et al. | 2009 | * | * | * | * |  | * | * | NA |
| rs35285779 | Pinarbasi et al. | 2010 | * | * |  |  | * | * | * | NA |
| rs35285779 | Gunes et al. | 2010 | * | * |  |  | ** | * | * | NA |
| rs35285779 | Bahl et al. | 2017 | * | * | * | * | ** | * | * | NA |
| rs35415678 | Gunes et al. | 2009 | * | * | * | * |  | * | * | NA |
| rs35415678 | Pinarbasi et al. | 2010 | * | * |  |  | * | * | * | NA |
| rs35415678 | Gunes et al. | 2010 | * | * |  |  | ** | * | * | NA |
| rs35415678 | Bahl et al. | 2017 | * | * | * | * | ** | * | * | NA |
| rs3923086 | Wang et al. | 2008 | * | * | * | * | ** | * | * | NA |
| rs3923086 | Filho et al. | 2011 | * | * |  |  | * | * | * | NA |
| rs3923086 | Alanazi et al. | 2013 | * | * | * | * | ** | * | * | NA |
| rs3923086 | Liu et al. | 2016 | * | * |  |  | ** | * | * | NA |
| rs3923086 | Parine et al. | 2019 | * | * | * | * | ** | * | * | NA |
| rs3923087 | Wang et al. | 2008 | * | * | * | * | ** | * | * | NA |
| rs3923087 | Filho et al. | 2011 | * | * |  |  | * | * | * | NA |
| rs3923087 | Mostowska et al. | 2013 | * |  |  |  | * | * | * | NA |
| rs3923087 | Alanazi et al. | 2013 | * | * | * | * | ** | * | * | NA |
| rs3923087 | Parine et al. | 2019 | * | * | * | * | ** | * | * | NA |
| rs4072245 | Gunes et al. | 2009 | * | * | * | * |  | * | * | NA |
| rs4072245 | Pinarbasi et al. | 2010 | * | * |  |  | * | * | * | NA |
| rs4072245 | Gunes et al. | 2010 | * | * |  |  | ** | * | * | NA |
| rs4791171 | Wang et al. | 2008 | * | * | * | * | ** | * | * | NA |
| rs4791171 | Filho et al. | 2011 | * | * |  |  | * | * | * | NA |
| rs4791171 | Alanazi et al. | 2013 | * | * | * | * | ** | * | * | NA |
| rs4791171 | Yadav et al. | 2015 | * | * | * | * | ** | * | * | NA |
| rs4791171 | Parine et al. | 2019 | * | * | * | * | ** | * | * | NA |
| rs7219582 | Gunes et al. | 2009 | * | * | * | * |  | * | * | NA |
| rs7219582 | Pinarbasi et al. | 2010 | * | * |  |  | * | * | * | NA |
| rs7219582 | Gunes et al. | 2010 | * | * |  |  | ** | * | * | NA |
| rs7219582 | Bahl et al. | 2017 | * | * | * | * | ** | * | * | NA |
| rs7224837 | Filho et al. | 2011 | * | * |  |  | * | * | * | NA |
| rs7224837 | Mostowska et al. | 2013 | * |  |  |  | * | * | * | NA |
| rs7224837 | Jeanne et al. | 2015 | * | * |  |  | ** | * | * | NA |
| rs9915936 | Gunes et al. | 2009 | * | * | * | * |  | * | * | NA |
| rs9915936 | Pinarbasi et al. | 2010 | * | * |  |  | * | * | * | NA |
| rs9915936 | Gunes et al. | 2010 | * | * |  |  | ** | * | * | NA |
| rs9915936 | Bahl et al. | 2017 | * | * | * | * | ** | * | * | NA |
| rs9915936 | Bahl et al. | 2017 | * | * | * | * | ** | * | * | NA |

This table identifies “high” quality choices with a “star”. A study can be awarded a maximum of 1 star for each numbered item within the Selection and Exposure categories. A maximum of 2 stars can be given for Comparability. *, Yes; NA, not applicable. (http://www.ohri.ca/programs/clinical epidemiology/oxford.htm).

**Supplementary table 2. Results of pooled analysis for *AXIN2* Polymorphism and cancer susceptibility.**

| **Polymorphism** | **Comparison** | **Subgroup** | **N** | **P_H_** | **P_Z_** | **P_Adjust_** | **OR & 95%CI (Random)** | **OR & 95%CI (Fixed)** |
| --- | --- | --- | --- | --- | --- | --- | --- | --- |
| rs2240307 | B vs. A | Overall | 6 | 0.363 | 0.29 | 1.000 | 1.141（0.917-1.42） | 1.103（0.92-1.323） |
| rs2240307 | BA vs. AA | Overall | 5 | 0.583 | 0.191 | 0.955 | 1.272（0.872-1.856） | 1.281（0.883-1.859） |
| rs2240307 | BB+BA vs. AA | Overall | 5 | 0.786 | 0.074 | 0.37 | 1.383（0.961-1.991） | 1.39（0.969-1.994） |
| rs2240307 | B vs. A | Asian | 5 | 0.871 | 0.021 | 0.105 | 1.463（1.054-2.033） | 1.467（1.058-2.035） |
| rs2240307 | BA vs. AA | Asian | 5 | 0.583 | 0.191 | 0.955 | 1.272（0.872-1.856） | 1.281（0.883-1.859） |
| rs2240307 | BB+BA vs. AA | Asian | 5 | 0.786 | 0.074 | 0.37 | 1.383（0.961-1.991） | 1.39（0.969-1.994） |
| rs2240307 | B vs. A | PB | 3 | 0.552 | 0.032 | 0.16 | 1.456（1.027-2.065） | 1.461（1.033-2.067） |
| rs2240307 | BA vs. AA | PB | 3 | 0.267 | 0.288 | 1.000 | 1.217（0.744-1.993） | 1.244（0.832-1.858） |
| rs2240307 | BB+BA vs. AA | PB | 3 | 0.443 | 0.113 | 0.565 | 1.36（0.919-2.013） | 1.369（0.929-2.017） |
| rs2240307 | B vs. A | HB | 3 | 0.661 | 0.919 | 1.000 | 0.988（0.797-1.226） | 0.989（0.798-1.226） |
| rs2240307 | BA vs. AA | HB | 2 | 0.829 | 0.396 | 1.000 | 1.535（0.57-4.135） | 1.536（0.571-4.134） |
| rs2240307 | BB+BA vs. AA | HB | 2 | 0.829 | 0.396 | 1.000 | 1.535（0.57-4.135） | 1.536（0.571-4.134） |
| rs2240307 | B vs. A | Lung Cancer | 3 | 0.552 | 0.032 | 0.16 | 1.456（1.027-2.065） | 1.461（1.033-2.067） |
| rs2240307 | BA vs. AA | Lung Cancer | 3 | 0.267 | 0.288 | 1.000 | 1.217（0.744-1.993） | 1.244（0.832-1.858） |
| rs2240307 | BB+BA vs. AA | Lung Cancer | 3 | 0.443 | 0.113 | 0.565 | 1.36（0.919-2.013） | 1.369（0.929-2.017） |
| rs35415678 | B vs. A | Overall | 4 | 0.761 | 0.759 | 1.000 | 0.95（0.674-1.337） | 0.948（0.674-1.333） |
| rs35415678 | BA vs. AA | Overall | 4 | 0.743 | 0.75 | 1.000 | 0.946（0.663-1.35） | 0.944（0.662-1.345） |
| rs35415678 | BB+BA vs. AA | Overall | 4 | 0.743 | 0.75 | 1.000 | 0.946（0.663-1.35） | 0.944（0.662-1.345） |
| rs35415678 | B vs. A | PB | 2 | 0.286 | 0.792 | 1.000 | 0.934（0.605-1.444） | 0.95（0.648-1.392） |
| rs35415678 | BA vs. AA | PB | 2 | 0.271 | 0.784 | 1.000 | 0.921（0.572-1.484） | 0.946（0.635-1.408） |
| rs35415678 | BB+BA vs. AA | PB | 2 | 0.271 | 0.784 | 1.000 | 0.921（0.572-1.484） | 0.946（0.635-1.408） |
| rs35415678 | B vs. A | HB | 2 | 0.863 | 0.874 | 1.000 | 0.941（0.443-1.997） | 0.941（0.443-1.997） |
| rs35415678 | BA vs. AA | HB | 2 | 0.86 | 0.87 | 1.000 | 0.937（0.43-2.042） | 0.937（0.43-2.042） |
| rs35415678 | BB+BA vs. AA | HB | 2 | 0.86 | 0.87 | 1.000 | 0.937（0.43-2.042） | 0.937（0.43-2.042） |
| rs3923086 | B vs. A | Overall | 5 | 0.026 | 0.609 | 1 | 0.939 (0.736-1.197) | 1.07 (0.957-1.196) |
| rs3923086 | BB vs. AA | Overall | 4 | 0.078 | 0.809 | 1 | 0.932（0.527-1.648） | 1.198（0.934-1.536） |
| rs3923086 | BA vs. AA | Overall | 4 | 0.071 | 0.318 | 1 | 0.803（0.521-1.236） | 1（0.824-1.213） |
| rs3923086 | BB+BA vs. AA | Overall | 4 | 0.02 | 0.318 | 1 | 0.781（0.481-1.268） | 1.045（0.871-1.253） |
| rs3923086 | BB vs. BA+ AA | Overall | 4 | 0.292 | 0.146 | 0.73 | 1.103（0.814-1.496） | 1.175（0.945-1.46） |
| rs3923086 | B vs. A | Asian | 3 | 0.155 | 0.058 | 0.29 | 0.738（0.499-1.09） | 0.777（0.599-1.009） |
| rs3923086 | BB vs. AA | Asian | 3 | 0.462 | 0.167 | 0.835 | 0.693（0.406-1.182） | 0.689（0.406-1.169） |
| rs3923086 | BA vs. AA | Asian | 3 | 0.31 | 0.054 | 0.27 | 0.658（0.418-1.036） | 0.67（0.445-1.007） |
| rs3923086 | BB+BA vs. AA | Asian | 3 | 0.212 | 0.038 | 0.19 | 0.632（0.382-1.045） | 0.667（0.455-0.977） |
| rs3923086 | BB vs. BA+ AA | Asian | 3 | 0.674 | 0.425 | 1 | 0.838（0.537-1.308） | 0.835（0.536-1.3） |
| rs3923086 | B vs. A | Caucasian | 3 | 0.005 | 0.901 | 1 | 1.015(0.807-1.276) | 1.006(0.918-1.103) |
| rs3923086 | B vs. A | PB | 3 | 0.056 | 0.864 | 1 | 0.975 (0.727-1.307) | 1.097 (0.969-1.241) |
| rs3923086 | BB vs. AA | PB | 3 | 0.053 | 0.914 | 1 | 0.697(0.262-1.854) | 0.96(0.74-1.246) |
| rs3923086 | BA vs. AA | PB | 3 | 0.211 | 0.677 | 1 | 0.928(0.703-1.225) | 0.982(0.893-1.079) |
| rs3923086 | BB+BA vs. AA | PB | 3 | 0.088 | 0.744 | 1 | 0.934（0.619-1.409） | 1.091（0.905-1.314） |
| rs3923086 | BB vs. BA+ AA | PB | 3 | 0.22 | 0.129 | 0.645 | 1.1（0.789-1.533） | 1.184（0.952-1.473） |
| rs3923086 | B vs. A | HB | 2 | 0.032 | 0.47 | 1 | 0.697(0.262-1.854) | 0.96(0.74-1.246) |
| rs3923086 | B vs. A | Breast Cancer | 3 | 0.023 | 0.845 | 1 | 0.953 (0.59-1.54) | 1.116 (0.979-1.271) |
| rs3923086 | BB vs. AA | Breast Cancer | 2 | 0.021 | 0.869 | 1 | 0.923（0.354-2.402） | 1.251（0.959-1.631） |
| rs3923086 | BA vs. AA | Breast Cancer | 2 | 0.097 | 0.721 | 1 | 0.893（0.48-1.661） | 1.066（0.863-1.316） |
| rs3923086 | BB+BA vs. AA | Breast Cancer | 2 | 0.038 | 0.728 | 1 | 0.877（0.419-1.837） | 1.12（0.918-1.366） |
| rs3923086 | BB vs. BA+ AA | Breast Cancer | 2 | 0.095 | 0.809 | 1 | 1.066（0.633-1.798） | 1.206（0.958-1.518） |
| rs3923086 | B vs. A | Y | 4 | 0.011 | 0.414 | 1 | 0.863（0.605-1.23） | 1.073（0.95-1.212） |
| rs3923086 | BB vs. AA | Y | 4 | 0.078 | 0.809 | 1 | 0.932（0.527-1.648） | 1.198（0.934-1.536） |
| rs3923086 | BA vs. AA | Y | 4 | 0.071 | 0.318 | 1 | 0.803（0.521-1.236） | 1（0.824-1.213） |
| rs3923086 | BB+BA vs. AA | Y | 4 | 0.02 | 0.318 | 1 | 0.781（0.481-1.268） | 1.045（0.871-1.253） |
| rs3923086 | BB vs. BA+ AA | Y | 4 | 0.292 | 0.146 | 0.73 | 1.103（0.814-1.496） | 1.175（0.945-1.46） |
| rs3923087 | B vs. A | Overall | 5 | <0.001 | 0.894 | 1 | **1.021 (0.751-1.388)** | 0.962 (0.856-1.083) |
| rs3923087 | BB vs. AA | Overall | 4 | 0.195 | 0.269 | 1 | 0.859（0.564-1.309） | **0.838（0.613-1.146）** |
| rs3923087 | BA vs. AA | Overall | 4 | 0.05 | 0.432 | 1 | **0.817（0.494-1.352）** | 0.819（0.61-1.099） |
| rs3923087 | BB+BA vs. AA | Overall | 4 | 0.046 | 0.39 | 1 | **0.81（0.501-1.309）** | 0.801（0.606-1.057） |
| rs3923087 | BB vs. BA+ AA | Overall | 4 | 0.503 | 0.089 | 0.445 | 0.868（0.739-1.021） | **0.869（0.739-1.022）** |
| rs3923087 | B vs. A | Asian | 2 | 0.018 | 0.721 | 1 | 0.888（0.463-1.704） | 0.916（0.699-1.201） |
| rs3923087 | BB vs. AA | Asian | 2 | 0.052 | 0.809 | 1 | 0.879（0.309-2.503） | 0.93（0.55-1.573） |
| rs3923087 | BA vs. AA | Asian | 2 | 0.011 | 0.488 | 1 | 0.67（0.216-2.077） | 0.699（0.453-1.079） |
| rs3923087 | BB+BA vs. AA | Asian | 2 | 0.007 | 0.576 | 1 | 0.728（0.239-2.213） | 0.758（0.506-1.134） |
| rs3923087 | BB vs. BA+ AA | Asian | 2 | 0.386 | 0.64 | 1 | 1.116（0.701-1.778） | 1.117（0.703-1.776） |
| rs3923087 | B vs. A | Caucasian | 3 | <0.001 | 0.638 | 1 | 1.107 (0.726-1.687) | 0.974 (0.854-1.109) |
| rs3923087 | BB vs. AA | Caucasian | 2 | 0.4 | 0.24 | 1 | 0.791（0.534-1.17） | 0.791（0.536-1.169） |
| rs3923087 | BA vs. AA | Caucasian | 2 | 0.507 | 0.751 | 1 | 0.937（0.626-1.401） | 0.937（0.627-1.4） |
| rs3923087 | BB+BA vs. AA | Caucasian | 2 | 0.427 | 0.38 | 1 | 0.841（0.572-1.238） | 0.842（0.573-1.237） |
| rs3923087 | BB vs. BA+ AA | Caucasian | 2 | 0.568 | 1 | 1 | 0.839（0.706-0.997） | 0.839（0.706-0.997） |
| rs3923087 | B vs. A | PB | 3 | 0.051 | 0.374 | 1 | 0.871 (0.642-1.181) | 0.859 (0.747-0.988) |
| rs3923087 | BB vs. AA | PB | 3 | 0.118 | 0.204 | 1 | 1.299(0.709-2.377) | 1.266(1.018-1.576) |
| rs3923087 | BA vs. AA | PB | 3 | 0.031 | 0.34 | 1 | 0.744（0.405-1.367） | 0.777（0.567-1.063） |
| rs3923087 | BB+BA vs. AA | PB | 3 | 0.027 | 0.333 | 1 | 0.749（0.418-1.343） | 0.766（0.57-1.03） |
| rs3923087 | BB vs. BA+ AA | PB | 3 | 0.328 | 0.095 | 0.475 | 0.875（0.697-1.1） | 0.856（0.714-1.027） |
| rs3923087 | B vs. A | HB | 2 | 0.006 | 0.397 | 1 | 1.299(0.709-2.377) | 1.266(1.018-1.576) |
| rs3923087 | B vs. A | Y | 4 | 0.095 | 0.289 | 1 | 0.891（0.721-1.102） | 0.876（0.772-0.995） |
| rs3923087 | BB vs. AA | Y | 4 | 0.195 | 0.269 | 1 | 0.859（0.564-1.309） | 0.838（0.613-1.146） |
| rs3923087 | BA vs. AA | Y | 4 | 0.05 | 0.432 | 1 | 0.817（0.494-1.352） | 0.819（0.61-1.099） |
| rs3923087 | BB+BA vs. AA | Y | 4 | 0.046 | 0.39 | 1 | 0.81（0.501-1.309） | 0.801（0.606-1.057） |
| rs3923087 | BB vs. BA+ AA | Y | 4 | 0.503 | 0.089 | 0.445 | 0.868（0.739-1.021） | 0.869（0.739-1.022） |
| rs4072245 | B vs. A | Overall | 3 | 0.184 | 0.761 | 1.000 | 0.925（0.556-1.538） | 0.942（0.643-1.381） |
| rs4072245 | BA vs. AA | Overall | 3 | 0.149 | 0.747 | 1.000 | 0.914（0.516-1.619） | 0.935（0.624-1.403） |
| rs4072245 | BB+BA vs. AA | Overall | 3 | 0.149 | 0.747 | 1.000 | 0.914（0.516-1.619） | 0.935（0.624-1.403） |
| rs4072245 | B vs. A | HB | 2 | 0.381 | 0.207 | 1.000 | 0.73（0.442-1.204） | 0.726（0.442-1.194） |
| rs4072245 | BA vs. AA | HB | 2 | 0.359 | 0.183 | 0.915 | 0.704（0.416-1.193） | 0.701（0.415-1.183） |
| rs4072245 | BB+BA vs. AA | HB | 2 | 0.359 | 0.183 | 0.915 | 0.704（0.416-1.193） | 0.701（0.415-1.183） |
| rs4791171 | B vs. A | Overall | 5 | 0.729 | 0.08 | 0.4 | 0.916（0.83-1.011） | 0.916（0.83-1.011） |
| rs4791171 | BB vs. AA | Overall | 4 | 0.327 | 0.111 | 0.555 | 0.83（0.628-1.095） | 0.817（0.638-1.047） |
| rs4791171 | BA vs. AA | Overall | 4 | 0.203 | 0.237 | 1.000 | 0.878（0.645-1.194） | 0.867（0.685-1.098） |
| rs4791171 | BB+BA vs. AA | Overall | 4 | 0.192 | 0.147 | 0.735 | 0.863（0.643-1.157） | 0.848（0.679-1.06） |
| rs4791171 | BB vs. BA+ AA | Overall | 4 | 0.928 | 0.205 | 1.000 | 0.905（0.775-1.056） | 0.905（0.775-1.056） |
| rs4791171 | B vs. A | Caucasian | 2 | 0.473 | 0.044 | 0.22 | 0.883（0.782-0.997） | 0.883（0.782-0.997） |
| rs4791171 | B vs. A | Asian | 3 | 0.787 | 0.858 | 1.000 | 0.985（0.83-1.168） | 0.985（0.83-1.168） |
| rs4791171 | BB vs. AA | Asian | 3 | 0.825 | 0.927 | 1.000 | 1.016（0.717-1.441） | 1.017（0.717-1.44） |
| rs4791171 | BA vs. AA | Asian | 3 | 0.324 | 0.904 | 1.000 | 1.011（0.72-1.419） | 1.02（0.745-1.395） |
| rs4791171 | BB+BA vs. AA | Asian | 3 | 0.423 | 0.969 | 1.000 | 1.006（0.748-1.353） | 1.006（0.749-1.35） |
| rs4791171 | BB vs. BA+ AA | Asian | 3 | 0.942 | 0.764 | 1.000 | 0.962（0.744-1.242） | 0.962（0.745-1.242） |
| rs4791171 | B vs. A | Breast Cancer | 2 | 0.952 | 0.031 | 0.155 | 0.856（0.743-0.986） | 0.856（0.743-0.986） |
| rs4791171 | BB vs. AA | Breast Cancer | 2 | 0.656 | 0.017 | 0.085 | 0.671（0.483-0.933） | 0.671（0.483-0.932） |
| rs4791171 | BA vs. AA | Breast Cancer | 2 | 0.844 | 0.022 | 0.11 | 0.687（0.499-0.947） | 0.687（0.499-0.947） |
| rs4791171 | BB+BA vs. AA | Breast Cancer | 2 | 0.945 | 0.012 | 0.06 | 0.676（0.499-0.916） | 0.676（0.499-0.916） |
| rs4791171 | BB vs. BA+ AA | Breast Cancer | 2 | 0.637 | 0.199 | 0.995 | 0.885（0.734-1.066） | 0.885（0.734-1.066） |
| rs4791171 | B vs. A | PB | 4 | 0.579 | 0.097 | 0.485 | 0.909（0.812-1.018） | 0.909（0.812-1.018） |
| rs4791171 | BB vs. AA | PB | 4 | 0.327 | 0.111 | 0.555 | 0.83（0.628-1.095） | 0.817（0.638-1.047） |
| rs4791171 | BA vs. AA | PB | 4 | 0.203 | 0.237 | 1.000 | 0.878（0.645-1.194） | 0.867（0.685-1.098） |
| rs4791171 | BB+BA vs. AA | PB | 4 | 0.192 | 0.147 | 0.735 | 0.863（0.643-1.157） | 0.848（0.679-1.06） |
| rs4791171 | BB vs. BA+ AA | PB | 4 | 0.928 | 0.205 | 1.000 | 0.905（0.775-1.056） | 0.905（0.775-1.056） |
| rs4791171 | B vs. A | Y | 4 | 0.579 | 0.097 | 0.485 | 0.909（0.812-1.018） | 0.909（0.812-1.018） |
| rs4791171 | BB vs. AA | Y | 4 | 0.327 | 0.111 | 0.555 | 0.83（0.628-1.095） | 0.817（0.638-1.047） |
| rs4791171 | BA vs. AA | Y | 4 | 0.203 | 0.237 | 1.000 | 0.878（0.645-1.194） | 0.867（0.685-1.098） |
| rs4791171 | BB+BA vs. AA | Y | 4 | 0.192 | 0.147 | 0.735 | 0.863（0.643-1.157） | 0.848（0.679-1.06） |
| rs4791171 | BB vs. BA+ AA | Y | 4 | 0.928 | 0.205 | 1.000 | 0.905（0.775-1.056） | 0.905（0.775-1.056） |
| rs7224837 | B vs. A | Overall | 3 | 0.354 | 0.049 | 0.245 | 0.845（0.709-1.007） | 0.843（0.711-0.999） |
| rs7224837 | BB vs. AA | Overall | 2 | 0.156 | 0.057 | 0.285 | 0.544（0.198-1.497） | 0.51（0.255-1.02） |
| rs7224837 | BA vs. AA | Overall | 2 | 0.316 | 0.376 | 1.000 | 0.91（0.738-1.123） | 0.91（0.738-1.122） |
| rs7224837 | BB+BA vs. AA | Overall | 2 | 0.22 | 0.183 | 0.915 | 0.89（0.682-1.161） | 0.871（0.711-1.067） |
| rs7224837 | BB vs. BA+ AA | Overall | 2 | 0.177 | 0.061 | 0.305 | 0.547（0.21-1.426） | 0.516（0.259-1.031） |

*P****_H_***: *P* value of Q test for heterogeneity test; *P_Z_:* *P* value of meta-analysis; *P_Adjust_*: Adjust *P_Z_* value by Bonferroni corrections, *P_Adjust_* = *P_Z_* * 5; P-B: Population based; HWE: Hardy Weinberg Equilibrium; Y: polymorphisms conformed to HWE in the control group; N: polymorphisms didn’t conform to HWE in the control group; * *P* value less than 0.05 was considered as statistically significant.

**Supplementary table 3. Details of the sensitivity analyses for *AXIN2* polymorphism and urinary cancer risk.**

| **Polymorphisms** | **Comparison** | **Study omitted** | **Estimate (95% Confident Interval)** | **Effect Model** |
| --- | --- | --- | --- | --- |
| rs11079571 | B vs. A | Wang et al. (2008) | 0.35127548(0.19104746-0.64588382) | Random |
|  |  | Alanazi et al. (2013) | 0.60373701(0.37237683-0.97884279) |  |
|  |  | Zhang et al. (2015) | 0.44628977(0.14957671-1.3315881) |  |
|  |  | Combined | 0.4594107(0.26579693-0.79405804) |  |
| rs11079571 | BB vs. AA | Wang et al. (2008) | 0.13549247(0.04506474-0.40737408) | Random |
|  |  | Alanazi et al. (2013) | 0.30289376(0.15995833-0.57355332) |  |
|  |  | Zhang et al. (2015) | 0.18224679(0.03172991-1.0467691) |  |
|  |  | Combined | 0.20006104(0.08525669-0.46945781) |  |
| rs11079571 | BA vs. AA | Wang et al. (2008) | 0.24573924(0.17430203-0.34645477) | Random |
|  |  | Alanazi et al. (2013) | 0.333312(0.14635891-0.75907165) |  |
|  |  | Zhang et al. (2015) | 0.4192819(0.25514141-0.68901908) |  |
|  |  | Combined | 0.32173897(0.19172905-0.53990756) |  |
| rs11079571 | BB+BA vs. AA | Wang et al. (2008) | 0.21630837(0.15740518-0.29725394) | Random |
|  |  | Alanazi et al. (2013) | 0.31072706(0.15548825-0.62095571) |  |
|  |  | Zhang et al. (2015) | 0.29408398(0.11782904-0.73399037) |  |
|  |  | Combined | 0.26503261(0.16227065-0.43287115) |  |
| rs11079571 | BB vs. BA+ AA | Wang et al. (2008) | 0.31115526(0.08301794-1.166225) | Random |
|  |  | Alanazi et al. (2013) | 0.69457412(0.53284711-0.90538776) |  |
|  |  | Zhang et al. (2015) | 0.35603923(0.07286067-1.7398126) |  |
|  |  | Combined | 0.43628589(0.21453297-0.88725466) |  |
| rs1133683 | B vs. A | Gunes et al. (2009) | 1.1312273(0.77569717-1.6497096) | Random |
|  |  | Pina et al. (2010) | 1.1384109(0.78197968-1.6573057) |  |
|  |  | Gunes et al. (2010) | 0.99901807(0.68730295-1.4521067) |  |
|  |  | Davoodi et al. (2015) | 0.96949095(0.68696666-1.3682071) |  |
|  |  | RR (2016) | 1.2117214(0.90358508-1.6249369) |  |
|  |  | Bahl et al. (2017) | 1.0281307(0.67292005-1.5708443) |  |
|  |  | Combined | 1.0761101(0.77325273-1.4975867) |  |
| rs1133683 | BB vs. AA | Gunes et al. (2009) | 0.24757689(0.08084814-0.7581414) | Random |
|  |  | Pina et al. (2010) | 0.35770458(0.15242277-0.83945823) |  |
|  |  | Gunes et al. (2010) | 0.18329884(0.07240166-0.46405655) |  |
|  |  | Davoodi et al. (2015) | 0.30017063(0.10710762-0.84123248) |  |
|  |  | RR (2016) | 0.28290132(0.09327751-0.85801131) |  |
|  |  | Bahl et al. (2017) | 0.21106209(0.06690793-0.6657986) |  |
|  |  | Combined | 0.25792128(0.10125741-0.656973) |  |
| rs1133683 | BA vs. AA | Gunes et al. (2009) | 2.4080429(1.158079-5.0071454) | Random |
|  |  | Pina et al. (2010) | 2.3623784(1.116344-4.9992042) |  |
|  |  | Gunes et al. (2010) | 1.8558295(0.82863975-4.1563334) |  |
|  |  | Davoodi et al. (2015) | 1.720202(0.80373716-3.68167) |  |
|  |  | RR (2016) | 2.5097125(1.2468687-5.05158) |  |
|  |  | Bahl et al. (2017) | 1.7669333(0.82991374-3.7619011) |  |
|  |  | Combined | 2.0791791(1.0476879-4.1262151) |  |
| rs1133683 | BB+BA vs. AA | Gunes et al. (2009) | 2.037858(0.96627921-4.2977896) | Random |
|  |  | Pina et al. (2010) | 2.0184402(0.95053399-4.286118) |  |
|  |  | Gunes et al. (2010) | 1.5670174(0.70288026-3.4935451) |  |
|  |  | Davoodi et al. (2015) | 1.4861413(0.68575501-3.2207074) |  |
|  |  | RR (2016) | 2.1855011(1.1050154-4.3224874) |  |
|  |  | Bahl et al. (2017) | 1.5123962(0.70569575-3.2412584) |  |
|  |  | Combined | 1.7796731(0.89518855-3.5380664) |  |
| rs1133683 | BB vs. BA+ AA | Gunes et al. (2009) | 0.14266765(0.06303893-0.3228808) | Random |
|  |  | Pina et al. (2010) | 0.2041785(0.10434245-0.39953887) |  |
|  |  | Gunes et al. (2010) | 0.12668145(0.06471357-0.247988) |  |
|  |  | Davoodi et al. (2015) | 0.2041785(0.10434245-0.39953887) |  |
|  |  | RR (2016) | 0.15357478(0.06164274-0.38261136) |  |
|  |  | Bahl et al. (2017) | 0.15214932(0.05715088-0.40505788) |  |
|  |  | Combined | 0.16197379(0.08007742-0.32762681) |  |
| rs2240307 | B vs. A | Gunes et al. (2009) | 1.2044318(0.92071593-1.5755738) | Fixed |
|  |  | Pinarbasi et al. (2010) | 1.1718642(0.90186447-1.5226963) |  |
|  |  | Gunes et al. (2010) | 1.1781811(0.89611894-1.5490251) |  |
|  |  | Filho et al. (2011) | 1.4633776(1.0535038-2.032716) |  |
|  |  | Han et al. (2016) | 1.0626719(0.85255581-1.324572) |  |
|  |  | Bahl et al. (2017) | 1.0488008(0.86544847-1.270998) |  |
|  |  | Combined | 1.1411126(0.91684035-1.420245) |  |
| rs2240307 | BA vs. AA | Gunes et al. (2009) | 1.324865(0.89381772-1.9637864) | Fixed |
|  |  | Pinarbasi et al. (2010) | 1.2518011(0.85076874-1.841871) |  |
|  |  | Gunes et al. (2010) | 1.2553389(0.84265703-1.8701272) |  |
|  |  | Han et al. (2016) | 1.5531957(0.95459163-2.5271716) |  |
|  |  | Bahl et al. (2017) | 1.0318569(0.63920921-1.6656967) |  |
|  |  | Combined | 1.27214(0.87194808-1.8560051) |  |
| rs2240307 | BB+BA vs. AA | Gunes et al. (2009) | 1.4453661(0.9899314-2.1103313) | Fixed |
|  |  | Pinarbasi et al. (2010) | 1.3671931(0.94255787-1.9831321) |  |
|  |  | Gunes et al. (2010) | 1.3780593(0.93962038-2.0210793) |  |
|  |  | Han et al. (2016) | 1.5531957(0.95459163-2.5271716) |  |
|  |  | Bahl et al. (2017) | 1.2003371(0.76350915-1.8870881) |  |
|  |  | Combined | 1.3829159(0.96065637-1.9907809) |  |
| rs2240308 | B vs. A | Kanzaki et al. (2006) | 0.95642084(0.84375417-1.084132) | Random |
|  |  | Kanzaki et al. (2006) | 0.94802296(0.83635181-1.0746046) |  |
|  |  | Kanzaki et al. (2006) | 0.96878678(0.85812825-1.0937151) |  |
|  |  | Gunes et al. (2009) | 0.96592796(0.85478604-1.0915209) |  |
|  |  | Gunes et al. (2010) | 0.95338565(0.84080184-1.0810446) |  |
|  |  | Fernandez-Rozadilla et al. (2010) | 0.937689(1.82361823-1.0675585) |  |
|  |  | Pinarbasi et al. (2010) | 0.94495147(0.83346617-1.0713491) |  |
|  |  | Naghibalhossaini et al. (2011) | 0.94441724(0.83220851-1.0717553) |  |
|  |  | Filho et al. (2011) | 0.94974756(0.83436471-1.0810865) |  |
|  |  | Mostowska et al. (2013) | 0.95474291(0.83989727-1.0852922) |  |
|  |  | Liu et al. (2014) | 0.96207815(0.84748083-1.0921715) |  |
|  |  | Ma et al. (2014) | 0.96476716(0.85349512-1.0905459) |  |
|  |  | Aristizabal-Pachon et al. (2015) | 0.91373521(0.823392871-0.0139899) |  |
|  |  | Yadav et al. (2015) | 0.95738161(0.84201342-1.0885569) |  |
|  |  | Rosales-Reynoso et al. (2016) | 0.92883623(0.82289255-1.0484197) |  |
|  |  | Kim et al. (2016) | 0.94818497(0.83347958-1.0786765) |  |
|  |  | Han et al. (2016) | 0.93196136(0.82487172-1.0529541) |  |
|  |  | Kim et al. (2016) | 0.93843466(0.82495213-1.0675281) |  |
|  |  | Liu et al. (2016) | 0.96118969(0.85048485-1.0863047) |  |
|  |  | Bahl et al. (2017) | 0.96448886(0.85131937-1.0927024) |  |
|  |  | Combined | 0.94947541(0.84103463-1.0718983) |  |
| rs2240308 | BB vs. AA | Kanzaki et al. (2006) | 0.8984074(0.78002828-1.0347521) | Random |
|  |  | Kanzaki et al. (2006) | 0.88462156(0.76650578-1.0209385) |  |
|  |  | Kanzaki et al. (2006) | 0.89657587(0.77731448-1.0341352) |  |
|  |  | Gunes et al. (2009) | 0.89842033(0.78036362-1.0343372) |  |
|  |  | Gunes et al. (2010) | 0.89424145(0.77561313-1.0310137) |  |
|  |  | Fernandez-Rozadilla et al. (2010) | 0.86598063(0.74821258-1.0022854) |  |
|  |  | Pinarbasi et al. (2010) | 0.8884995(0.76992333-1.0253376) |  |
|  |  | Naghibalhossaini et al. (2011) | 0.88368183(0.76467258-1.0212131) |  |
|  |  | Mostowska et al. (2013) | 0.88944405(0.76837063-1.0295953) |  |
|  |  | Liu et al. (2014) | 0.89992511(0.77708828-1.0421792) |  |
|  |  | Ma et al. (2014) | 0.92093271(0.81453264-1.0412315) |  |
|  |  | Aristizabal-Pachon et al. (2015) | 0.86799508(0.76745939-0.98170072) |  |
|  |  | Yadav et al. (2015) | 0.88984764(0.76718354-1.0321244) |  |
|  |  | Rosales-Reynoso et al. (2016) | 0.87063116(0.75954354-0.99796605) |  |
|  |  | Kim et al. (2016) | 0.87585497(0.75516123-1.0158386) |  |
|  |  | Han et al. (2016) | 0.87975889(0.76206893-1.0156243) |  |
|  |  | Kim et al. (2016) | 0.86902267(0.74968362-1.0073588) |  |
|  |  | Liu et al. (2016) | 0.89901942(0.78292668-1.0323266) |  |
|  |  | Bahl et al. (2017) | 0.88832349(0.76653492-1.0294621) |  |
|  |  | Combined | 0.88717611(0.77278665-1.0184977) |  |
| rs2240308 | BA vs. AA | Kanzaki et al. (2006) | 0.9626407(0.72604853-1.2763294) | Random |
|  |  | Kanzaki et al. (2006) | 0.95037419(0.71775603-1.2583817) |  |
|  |  | Kanzaki et al. (2006) | 1.0025482(0.76841092-1.308028) |  |
|  |  | Gunes et al. (2009) | 0.99203849(0.75589138-1.30196) |  |
|  |  | Gunes et al. (2010) | 0.95993644(0.72398478-1.2727864) |  |
|  |  | Fernandez-Rozadilla et al. (2010) | 0.93152595(0.693793-1.2507197) |  |
|  |  | Pinarbasi et al. (2010) | 0.94165039(0.7102192-1.2484955) |  |
|  |  | Naghibalhossaini et al. (2011) | 0.94136971(0.70910424-1.2497133) |  |
|  |  | Mostowska et al. (2013) | 0.96959323(0.72684413-1.2934148) |  |
|  |  | Liu et al. (2014) | 0.98340511(0.73950118-1.3077539) |  |
|  |  | Ma et al. (2014) | 0.96083134(0.72592151-1.2717586) |  |
|  |  | Aristizabal-Pachon et al. (2015) | 0.87930191(0.69690961-1.1094291) |  |
|  |  | Yadav et al. (2015) | 0.97353905(0.72831738-1.3013259) |  |
|  |  | Rosales-Reynoso et al. (2016) | 0.9009338(0.68898362-1.1780857) |  |
|  |  | Kim et al. (2016) | 0.95883369(0.71963751-1.2775348) |  |
|  |  | Han et al. (2016) | 0.90933651(0.6928845-1.1934065) |  |
|  |  | Kim et al. (2016) | 0.93315178(0.697025-1.2492697) |  |
|  |  | Liu et al. (2016) | 0.97129172(0.7393927-1.2759223) |  |
|  |  | Bahl et al. (2017) | 0.99134976(0.75018418-1.3100441) |  |
|  |  | Combined | 0.9520069(0.72624268-1.2479536) |  |
| rs2240308 | BB+BA vs. AA | Kanzaki et al. (2006) | 0.90694225(0.7687189-1.0700195) | Random |
|  |  | Kanzaki et al. (2006) | 0.89298749(0.75632346-1.054346) |  |
|  |  | Kanzaki et al. (2006) | 0.91334069(0.77559853-1.0755452) |  |
|  |  | Gunes et al. (2009) | 0.91306633(0.77610159-1.0742024) |  |
|  |  | Gunes et al. (2010) | 0.90300816(0.76506948-1.0658166) |  |
|  |  | Fernandez-Rozadilla et al. (2010) | 0.87617713(0.73919708-1.0385408) |  |
|  |  | Pinarbasi et al. (2010) | 0.89388329(0.7568627-1.0557098) |  |
|  |  | Naghibalhossaini et al. (2011) | 0.89058542(0.75326443-1.0529402) |  |
|  |  | Mostowska et al. (2013) | 0.90097678(0.76070625-1.0671124) |  |
|  |  | Liu et al. (2014) | 0.90997374(0.76809645-1.0780576) |  |
|  |  | Ma et al. (2014) | 0.92451578(0.7912401-1.0802402) |  |
|  |  | Aristizabal-Pachon et al. (2015) | 0.85852116(0.74616325-0.98779809) |  |
|  |  | Yadav et al. (2015) | 0.90250766(0.76046914-1.0710757) |  |
|  |  | Rosales-Reynoso et al. (2016) | 0.87066114(0.74319351-1.0199912) |  |
|  |  | Kim et al. (2016) | 0.88831878(0.74775791-1.0553018) |  |
|  |  | Han et al. (2016) | 0.87875581(0.74544871-1.035902) |  |
|  |  | Kim et al. (2016) | 0.87857592(0.74020839-1.0428085) |  |
|  |  | Liu et al. (2016) | 0.91083968(0.77528858-1.0700905) |  |
|  |  | Bahl et al. (2017) | 0.9066(0.76587188-1.0731868) |  |
|  |  | Combined | 0.89534871(0.76278423-1.0509516) |  |
| rs2240308 | BB vs. BA+ AA | Kanzaki et al. (2006) | 1.0084364(0.8062309-1.2613559) | Random |
|  |  | Kanzaki et al. (2006) | 1.0045896(0.80424017-1.2548491) |  |
|  |  | Kanzaki et al. (2006) | 1.0439119(0.84774715-1.2854685) |  |
|  |  | Gunes et al. (2009) | 1.0344512(0.83388978-1.2832503) |  |
|  |  | Gunes et al. (2010) | 1.0065057(0.80453289-1.2591826) |  |
|  |  | Fernandez-Rozadilla et al. (2010) | 0.99189723(0.78265262-1.257084) |  |
|  |  | Pinarbasi et al. (2010) | 0.99185342(0.79366529-1.2395315) |  |
|  |  | Naghibalhossaini et al. (2011) | 0.99489313(0.79471272-1.245497) |  |
|  |  | Mostowska et al. (2013) | 1.0208472(0.81096089-1.2850546) |  |
|  |  | Liu et al. (2014) | 1.033174(0.82379001-1.2957776) |  |
|  |  | Ma et al. (2014) | 0.99913359(0.80043519-1.2471565) |  |
|  |  | Aristizabal-Pachon et al. (2015) | 0.95253932(0.79181582-1.1458865) |  |
|  |  | Yadav et al. (2015) | 1.0261763(0.8150984-1.2919151) |  |
|  |  | Rosales-Reynoso et al. (2016) | 0.96900088(0.78016025-1.2035511) |  |
|  |  | Kim et al. (2016) | 1.0160731(0.81012344-1.2743793) |  |
|  |  | Han et al. (2016) | 0.96834505(0.78275722-1.1979347) |  |
|  |  | Kim et al. (2016) | 0.99121934(0.78633368-1.2494897) |  |
|  |  | Liu et al. (2016) | 1.0162197(0.81808066-1.2623481) |  |
|  |  | Bahl et al. (2017) | 1.0462639(0.84252566-1.2992698) |  |
|  |  | Combined | 1.0050939(0.81097961-1.245671) |  |
| rs35285779 | B vs. A | Gunes et al. (2009) | 0.61922693(0.36878413-1.0397465) | Random |
|  |  | Pinarbasi et al. (2010) | 0.7239607(0.55443102-0.94532782) |  |
|  |  | Gunes et al. (2010) | 0.54949766(0.32170945-0.93857253) |  |
|  |  | Bahl et al. (2017) | 0.52370107(0.33171913-0.8267923) |  |
|  |  | Combined | 0.60316899(0.40937354-0.88870627) |  |
| rs35285779 | BB vs. AA | Gunes et al. (2009) | 0.3941884(0.16379215-0.94866878) | Fixed |
|  |  | Pinarbasi et al. (2010) | 0.49822918(0.22456379-1.1053977) |  |
|  |  | Gunes et al. (2010) | 0.27957892(0.10559713-0.74021316) |  |
|  |  | Bahl et al. (2017) | 0.31529474(0.14012468-0.70944518) |  |
|  |  | Combined | 0.36769129(0.17568744-0.76953073) |  |
| rs35285779 | BA vs. AA | Gunes et al. (2009) | 0.70627636(0.51519561-0.96822697) | Fixed |
|  |  | Pinarbasi et al. (2010) | 0.75852132(0.55461746-1.03739) |  |
|  |  | Gunes et al. (2010) | 0.65452158(0.47590956-0.90016794) |  |
|  |  | Bahl et al. (2017) | 0.59651279(0.40712643-0.87399757) |  |
|  |  | Combined | 0.68397316(0.51447117-0.90932071) |  |
| rs35285779 | BB+BA vs. AA | Gunes et al. (2009) | 0.66792369(0.49272349-0.90542072) | Fixed |
|  |  | Pinarbasi et al. (2010) | 0.72546494(0.53758025-0.97901541) |  |
|  |  | Gunes et al. (2010) | 0.60910505(0.44763508-0.82882005) |  |
|  |  | Bahl et al. (2017) | 0.53384024(0.37246957-0.76512396) |  |
|  |  | Combined | 0.63872865(0.4861578-0.83918079) |  |
| rs35285779 | BB vs. BA+ AA | Gunes et al. (2009) | 0.43282738(0.17982139-1.041809) | Fixed |
|  |  | Pinarbasi et al. (2010) | 0.53940481(0.24448335-1.1900914) |  |
|  |  | Gunes et al. (2010) | 0.317498(0.11965642-0.84245354) |  |
|  |  | Bahl et al. (2017) | 0.35458216(0.15808812-0.79530656) |  |
|  |  | Combined | 0.40803971(0.19527046-0.85264515) |  |
| rs35415678 | B vs. A | Gunes et al. (2009) | 1.0292358(0.69817883-1.517271) | Fixed |
|  |  | Pinarbasi et al. (2010) | 0.94037181(0.65791714-1.3440889) |  |
|  |  | Gunes et al. (2010) | 0.95023489(0.64092606-1.4088153) |  |
|  |  | Bahl et al. (2017) | 0.77437657(0.43228188-1.3871946) |  |
|  |  | Combined | 0.94398508(0.66236787-1.3453368) |  |
| rs35415678 | BA vs. AA | Gunes et al. (2009) | 1.0292677(0.6981101-1.517514) | Fixed |
|  |  | Pinarbasi et al. (2010) | 0.94211203(0.65818846-1.3485121) |  |
|  |  | Gunes et al. (2010) | 0.95248121(0.64119923-1.4148808) |  |
|  |  | Bahl et al. (2017) | 0.77593005(0.43200099-1.3936713) |  |
|  |  | Combined | 0.94573899(0.66266285-1.3497395) |  |
| rs35415678 | BB+BA vs. AA | Gunes et al. (2009) | 1.0292677(0.6981101-1.517514) | Fixed |
|  |  | Pinarbasi et al. (2010) | 0.94211203(0.65818846-1.3485121) |  |
|  |  | Gunes et al. (2010) | 0.95248121(0.64119923-1.4148808) |  |
|  |  | Bahl et al. (2017) | 0.77593005(0.43200099-1.3936713) |  |
|  |  | Combined | 0.94573899(0.66266285-1.3497395) |  |
| rs3923086 | B vs. A | Wang et al. (2008) | 0.8426226(0.62493931-1.1361309) | Random |
|  |  | Filho et al. (2011) | 0.86264423(0.60508293-1.2298398) |  |
|  |  | Alanazi et al. (2013) | 1.0133923(0.79871206-1.2857749) |  |
|  |  | Liu et al. (2016) | 1.0202295(0.84255514-1.235371) |  |
|  |  | Parine et al. (2019) | 0.91370181(0.67237086-1.2416526) |  |
|  |  | Combined | 0.93856528(0.73598482-1.1969062) |  |
| rs3923086 | BB vs. AA | Wang et al. (2008) | 0.69327837(0.40649-1.1824026) | Random |
|  |  | Alanazi et al. (2013) | 1.3166702(0.99802947-1.7370431) |  |
|  |  | Liu et al. (2016) | 0.96837449(0.53862053-1.74102) |  |
|  |  | Parine et al. (2019) | 0.84463131(0.34312049-2.079159) |  |
|  |  | Combined | 0.93202139(0.52698329-1.6483708) |  |
| rs3923086 | BA vs. AA | Wang et al. (2008) | 0.6579957(0.41803056-1.0357097) | Random |
|  |  | Alanazi et al. (2013) | 0.8690083(0.53941405-1.399992) |  |
|  |  | Liu et al. (2016) | 0.95754713(0.6871267-1.3343922) |  |
|  |  | Parine et al. (2019) | 0.71120125(0.36295471-1.3935822) |  |
|  |  | Combined | 0.80263452(0.52123047-1.2359641) |  |
| rs3923086 | BB+BA vs. AA | Wang et al. (2008) | 0.63205403(0.38222668-1.045171) | Random |
|  |  | Alanazi et al. (2013) | 0.8668136(0.51210517-1.4672098) |  |
|  |  | Liu et al. (2016) | 0.93365592(0.6187672-1.4087906) |  |
|  |  | Parine et al. (2019) | 0.68774682(0.3230812-1.4640149) |  |
|  |  | Combined | 0.7812346(0.48114958-1.2684777) |  |
| rs3923086 | BB vs. BA+ AA | Wang et al. (2008) | 0.83502203(0.53615487-1.3004858) | Fixed |
|  |  | Alanazi et al. (2013) | 1.2577978(0.99547553-1.5892458) |  |
|  |  | Liu et al. (2016) | 1.1840528(0.95205683-1.4725814) |  |
|  |  | Parine et al. (2019) | 1.1956643(0.95065522-1.5038188) |  |
|  |  | Combined | 1.1748639(0.9453547-1.4600924) |  |
| rs3923087 | B vs. A | Wang et al. (2008) | 1.0824806(0.72269055-1.6213915) | Random |
|  |  | Filho et al. (2011) | 0.89143356(0.72080098-1.1024594) |  |
|  |  | Mostowska et al. (2013) | 1.0387108(0.69227814-1.5585066) |  |
|  |  | Alanazi et al. (2013) | 1.1296496(0.80940697-1.5765964) |  |
|  |  | Parine et al. (2019) | 0.97831044(0.67963575-1.4082416) |  |
|  |  | Combined | 1.021174(0.75105813-1.3884364) |  |
| rs3923087 | BB vs. AA | Wang et al. (2008) | 0.97232652(0.62280357-1.5180049) | Fixed |
|  |  | Mostowska et al. (2013) | 0.8031432(0.57273269-1.1262479) |  |
|  |  | Alanazi et al. (2013) | 0.91552949(0.65142101-1.2867165) |  |
|  |  | Parine et al. (2019) | 0.72772509(0.51216614-1.0340079) |  |
|  |  | Combined | 0.83803073(0.61267783-1.1462721) |  |
| rs3923087 | BA vs. AA | Wang et al. (2008) | 0.80028373(0.36284795-1.7650754) | Random |
|  |  | Mostowska et al. (2013) | 0.74371469(0.40470675-1.3666972) |  |
|  |  | Alanazi et al. (2013) | 1.0073948(0.72123855-1.4070854) |  |
|  |  | Parine et al. (2019) | 0.71826309(0.37941062-1.3597455) |  |
|  |  | Combined | 0.81740296(0.49432316-1.3516413) |  |
| rs3923087 | BB+BA vs. AA | Wang et al. (2008) | 0.83037961(0.38990349-1.7684641) | Random |
|  |  | Mostowska et al. (2013) | 0.74929953(0.41791308-1.3434606) |  |
|  |  | Alanazi et al. (2013) | 0.96599692(0.69785273-1.3371733) |  |
|  |  | Parine et al. (2019) | 0.68689185(0.40744829-1.1579884) |  |
|  |  | Combined | 0.81015464(0.50123714-1.3094611) |  |
| rs3923087 | BB vs. BA+ AA | Wang et al. (2008) | 0.98775226(0.74486989-1.3098322) | Fixed |
|  |  | Mostowska et al. (2013) | 0.85641414(0.71405512-1.0271548) |  |
|  |  | Alanazi et al. (2013) | 0.86848497(0.7355125-1.0254973) |  |
|  |  | Parine et al. (2019) | 0.84090877(0.71078223-0.99485832) |  |
|  |  | Combined | 0.86888161(0.73900455-1.021584) |  |
| rs4072245 | B vs. A | Gunes et al.(2009) | 0.72599518(0.44153789-1.1937118) | Fixed |
|  |  | Pinarbasi et al. (2010) | 1.1408451(0.72713792-1.789932) |  |
|  |  | Gunes et al.(2010) | 0.96866149(0.6082412-1.542653) |  |
|  |  | Combined | 0.94231925(0.64308511-1.3807901) |  |
| rs4072245 | BA vs. AA | Gunes et al.(2009) | 0.70078337(0.41514558-1.182952) | Fixed |
|  |  | Pinarbasi et al. (2010) | 1.1612903(0.71878189-1.8762232) |  |
|  |  | Gunes et al.(2010) | 0.96473622(0.58858687-1.5812719) |  |
|  |  | Combined | 0.93539704(0.6238376-1.4025567) |  |
| rs4072245 | BB+BA vs. AA | Gunes et al.(2009) | 0.70078337(0.41514558-1.182952) | Fixed |
|  |  | Pinarbasi et al. (2010) | 1.1612903(0.71878189-1.8762232) |  |
|  |  | Gunes et al.(2010) | 0.96473622(0.58858687-1.5812719) |  |
|  |  | Combined | 0.93539704(0.6238376-1.4025567) |  |
| rs4791171 | B vs. A | Wang et al. (2008) | 0.96508819(0.84680468-1.0998938) | Fixed |
|  |  | Filho et al. (2011) | 0.90920347(0.8124212-1.0175152) |  |
|  |  | Alanazi et al. (2013) | 0.91886997(0.83021122-1.0169966) |  |
|  |  | Yadav et al. (2015) | 0.89530057(0.80152076-1.0000528) |  |
|  |  | Parine et al. (2019) | 0.90659684(0.81842321-1.00427) |  |
|  |  | Combined | 0.91581507(0.82986293-1.0106696) |  |
| rs4791171 | BB vs. AA | Wang et al. (2008) | 1.016516(0.71740794-1.4403307) | Fixed |
|  |  | Alanazi et al. (2013) | 0.81899583(0.63173544-1.0617644) |  |
|  |  | Yadav et al. (2015) | 0.72911704(0.54158425-0.98158616) |  |
|  |  | Parine et al. (2019) | 0.7867521(0.60372776-1.0252616) |  |
|  |  | Combined | 0.81723725(0.63781023-1.0471402) |  |
| rs4791171 | BA vs. AA | Wang et al. (2008) | 1.0195427(0.74517524-1.3949299) | Fixed |
|  |  | Alanazi et al. (2013) | 0.903265(0.702007-1.1622216) |  |
|  |  | Yadav et al. (2015) | 0.76311678(0.57643622-1.0102544) |  |
|  |  | Parine et al. (2019) | 0.83011091(0.64155948-1.0740768) |  |
|  |  | Combined | 0.86722728(0.6848999-1.0980921) |  |
| rs4791171 | BB+BA vs. AA | Wang et al. (2008) | 1.0057636(0.74943471-1.3497647) | Fixed |
|  |  | Alanazi et al. (2013) | 0.87325239(0.68852538-1.1075405) |  |
|  |  | Yadav et al. (2015) | 0.75544333(0.5796814-0.98449707) |  |
|  |  | Parine et al. (2019) | 0.80765349(0.63294536-1.0305853) |  |
|  |  | Combined | 0.84827181(0.67901516-1.0597187) |  |
| rs4791171 | BB vs. BA+ AA | Wang et al. (2008) | 0.96156269(0.74460816-1.2417307) | Fixed |
|  |  | Alanazi et al. (2013) | 0.89859551(0.76714689-1.0525674) |  |
|  |  | Yadav et al. (2015) | 0.89495069(0.74823344-1.070437) |  |
|  |  | Parine et al. (2019) | 0.89791197(0.76558024-1.0531174) |  |
|  |  | Combined | 0.90490022(0.77541312-1.0560105) |  |
| rs7210356 | BB vs. BA+ AA | Wang et al. (2008) | 0.44444445(0.03905948-5.0571809) | Fixed |
|  |  | Liu et al. (2016) | 0.88703585(0.69801974-1.1272354) |  |
|  |  | Combined | 0.88071582(0.693936-1.1177693) |  |
| rs7219582 | B vs. A | Gunes et al. (2009) | 0.82181019(0.65832263-1.0258981) | Fixed |
|  |  | Pinarbasi et al. (2010) | 0.82301849(0.65913051-1.0276561) |  |
|  |  | Gunes et al. (2010) | 0.78712463(0.6290006-0.98499936) |  |
|  |  | Bahl et al. (2017) | 1.2201666(0.57385242-2.5944064) |  |
|  |  | Combined | 0.82011918(0.65852385-1.0213684) |  |
| rs7219582 | BA vs. AA | Gunes et al. (2009) | 0.78143829(0.23886809-2.5564144) | Random |
|  |  | Pinarbasi et al. (2010) | 0.7956475(0.23769069-2.6633561) |  |
|  |  | Gunes et al. (2010) | 0.4100545(0.27958265-0.60141325) |  |
|  |  | Bahl et al. (2017) | 1.1973982(0.52787024-2.7161267) |  |
|  |  | Combined | 0.75002423(0.30034786-1.8729494) |  |
| rs7219582 | BB+BA vs. AA | Gunes et al. (2009) | 0.79360676(0.2519601-2.4996486) | Random |
|  |  | Pinarbasi et al. (2010) | 0.80804408(0.25089076-2.602468) |  |
|  |  | Gunes et al. (2010) | 0.42892346(0.29272309-0.62849611) |  |
|  |  | Bahl et al. (2017) | 1.1973982(0.52787024-2.7161267) |  |
|  |  | Combined | 0.7578873(0.31335251-1.8330575) |  |
| rs7224837 | B vs. A | Filho et al. (2011) | 0.8761431(0.66139311-1.160621) | Fixed |
|  |  | Mostowska et al. (2013) | 0.7844407(0.6444695-0.95481199) |  |
|  |  | Jeanne et al. (2015) | 0.95004159(0.72521883-1.244561) |  |
|  |  | Combined | 0.84488376(0.70919593-1.0065322) |  |
| rs7224837 | BB vs. AA | Mostowska et al. (2013) | 0.33655071(0.13183117-0.85917759) | Fixed |
|  |  | Jeanne et al. (2015) | 0.94565219(0.32161435-2.780529) |  |
|  |  | Combined | 0.5101752(0.25529047-1.0195395) |  |
| rs7224837 | BA vs. AA | Mostowska et al. (2013) | 0.85199773(0.66641641-1.089259) | Fixed |
|  |  | Jeanne et al. (2015) | 1.0832824(0.72627002-1.615791) |  |
|  |  | Combined | 0.9098999(0.73810227-1.1216844) |  |
| rs7224837 | BB+BA vs. AA | Mostowska et al. (2013) | 0.804887(0.63353819-1.0225793) | Fixed |
|  |  | Jeanne et al. (2015) | 1.0693451(0.72714603-1.572585) |  |
|  |  | Combined | 0.87097109(0.71077345-1.0672749) |  |
| rs7224837 | BB vs. BA+ AA | Mostowska et al. (2013) | 0.34762713(0.13634956-0.88628536) | Fixed |
|  |  | Jeanne et al. (2015) | 0.92567569(0.31650153-2.7073343) |  |
|  |  | Combined | 0.51644234(0.25872935-1.030856) |  |
| rs9915936 | B vs. A | Gunes et al. (2009) | 0.70244366(0.49424112-0.99835294) | Fixed |
|  |  | Pinarbasi et al. (2010) | 0.67760569(0.47998631-0.95658875) |  |
|  |  | Gunes et al. (2010) | 0.70244366(0.49424112-0.99835294) |  |
|  |  | Bahl et al. (2017) | 0.80963731(0.47370544-1.3837979) |  |
|  |  | Combined | 0.7071777(0.51002661-0.98053767) |  |
| rs9915936 | BA vs. AA | Gunes et al. (2009) | 0.61684728(0.41490498-0.91707885) | Fixed |
|  |  | Pinarbasi et al. (2010) | 0.59192044(0.40152732-0.87259269) |  |
|  |  | Gunes et al. (2010) | 0.61684728(0.41490498-0.91707885) |  |
|  |  | Bahl et al. (2017) | 0.80040222(0.46168026-1.3876351) |  |
|  |  | Combined | 0.6327778(0.43989738-0.91022989) |  |
| rs9915936 | BB+BA vs. AA | Gunes et al. (2009) | 0.65071833(0.44511005-0.95130259) | Fixed |
|  |  | Pinarbasi et al. (2010) | 0.62503511(0.43079701-0.90685147) |  |
|  |  | Gunes et al. (2010) | 0.65071833(0.44511005-0.95130259) |  |
|  |  | Bahl et al. (2017) | 0.80040222(0.46168026-1.3876351) |  |
|  |  | Combined | 0.66116906(0.46565778-0.93876781) |  |

**Supplementary table 4. *P* values of the Egger’s test for *AXIN2* polymorphism.**

| **Polymorphisms** | **Subgroup** | **Egger’s test P > |t|** |
| --- | --- | --- |
| rs11079571 | Overall | 0.395 |
| rs1133683 | Overall | 0.563 |
|  | Asians | 0.783 |
|  | PB | 0.601 |
|  | Y | 0.32 |
| rs2240307 | Overall | 0.258 |
|  | Asians | 0.663 |
|  | PB | 0.566 |
|  | Lung Cancer | 0.566 |
|  | Y | 0.663 |
| rs2240308 | Overall | 0.686 |
|  | Asians | 0.538 |
|  | Caucasians | 0.389 |
|  | PB | 0.412 |
|  | HB | 0.012 |
|  | Colorectal Cancer | 0.861 |
|  | Lung Cancer | 0.725 |
|  | Y | 0.332 |
|  | N | 0.269 |
| rs35285779 | Overall | 0.068 |
| rs35415678 | Overall | 0.641 |
| rs3923086 | Overall | 0.317 |
|  | Asian | 0.285 |
|  | Caucasian | 0.686 |
|  | PB | 0.78 |
|  | Breast Cancer | 0.841 |
|  | Y | 0.035 |
| rs3923087 | Overall | 0.53 |
|  | Caucasian | 0.388 |
|  | PB | 0.93 |
|  | Y | 0.821 |
| rs4072245 | Overall | 0.085 |
| rs4791171 | Overall | 0.391 |
|  | Asians | 0.684 |
|  | PB | 0.487 |
|  | Y | 0.487 |
| rs7210356 | Overall | 0.701 |
| rs7219582 | Overall | 0.537 |
|  | Y | 0.129 |
| rs7224837 | Overall | 0.689 |
| rs9915936 | Overall | 0.159 |
