# Supplementary material for: New insights of the correlation between AXIN2 polymorphism and cancer risk and susceptibility: evidence from 72 studies
Source: BMC Cancer. 2021 Apr 1;21:353. doi: 10.1186/s12885-021-08092-0 (PMC8017882; doi:10.1186/s12885-021-08092-0)
Supplement: Supplementary file 5 — Additional file 5 : Figure S4. Meta-analysis ofAXIN2-rs35285779 polymorphism and overall cancer risk in 5 genetic models. [file 12885_2021_8092_MOESM5_ESM.pdf]

Fig.S4 Meta-analysis of AXIN2-rs35285779 polymorphism and overall cancer risk in 5 genetic models.

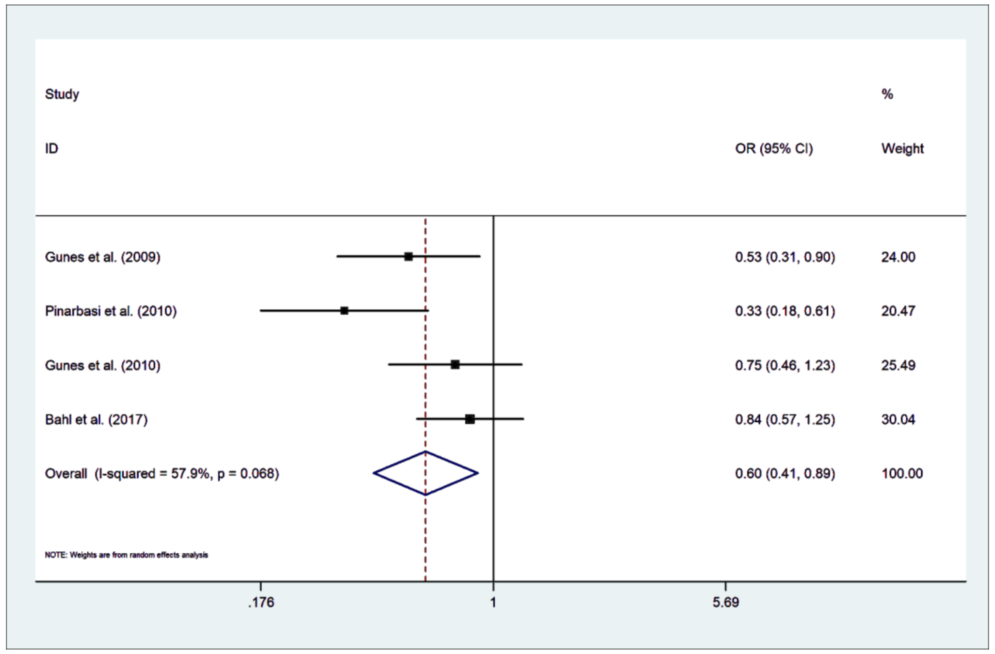

B VS A

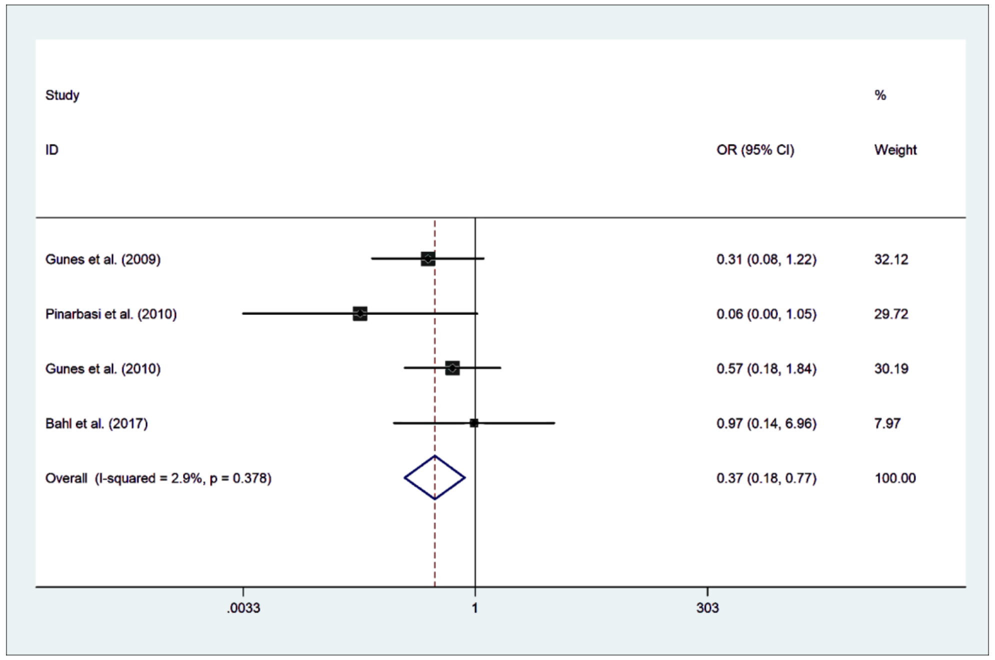

BB VS AA

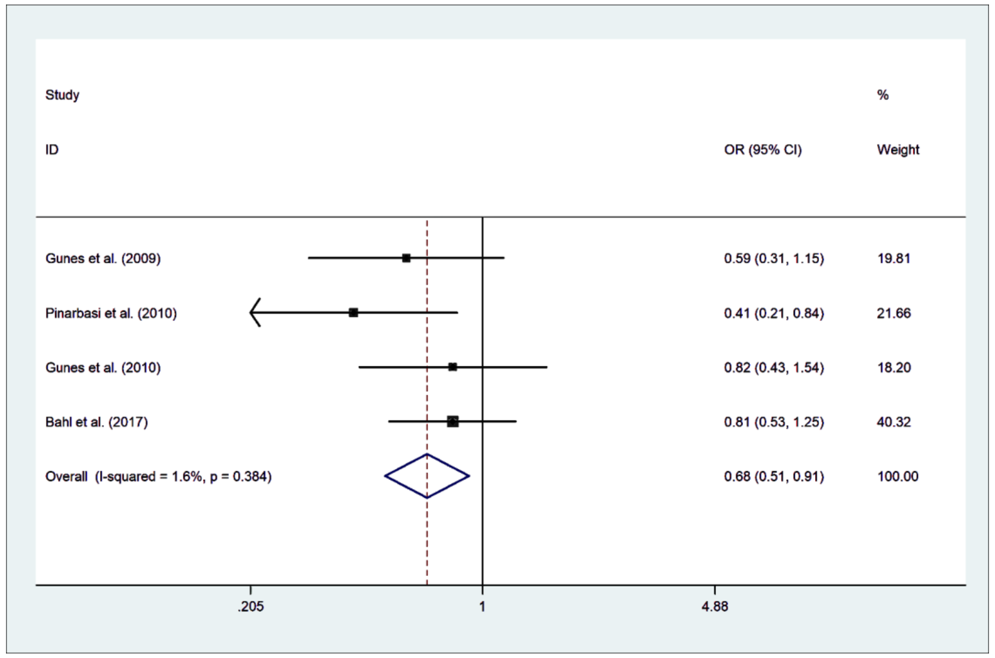

BA VS AA

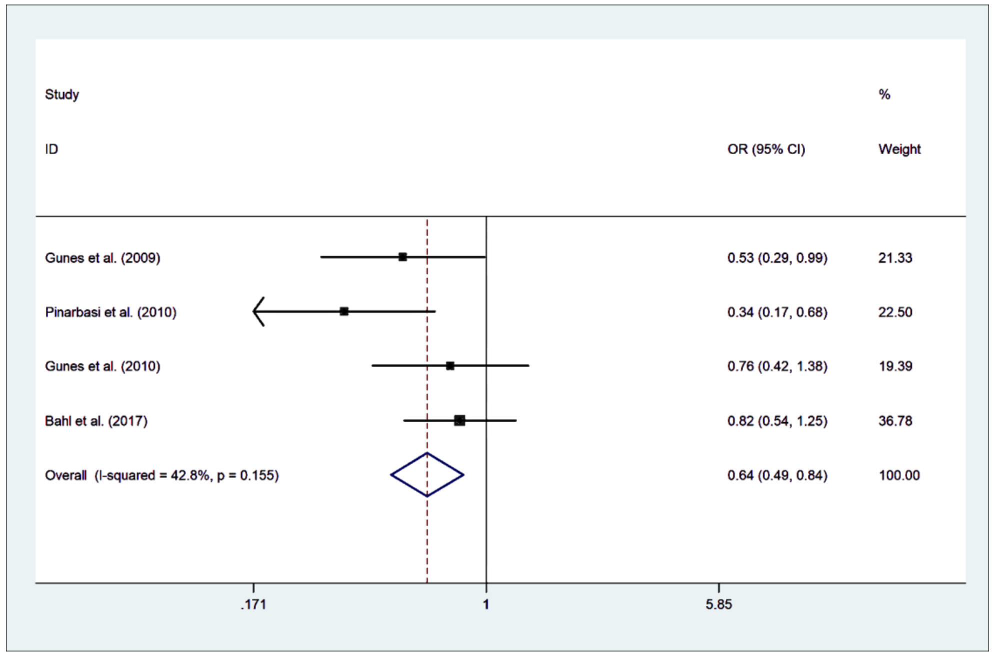

BB+BA VS AA

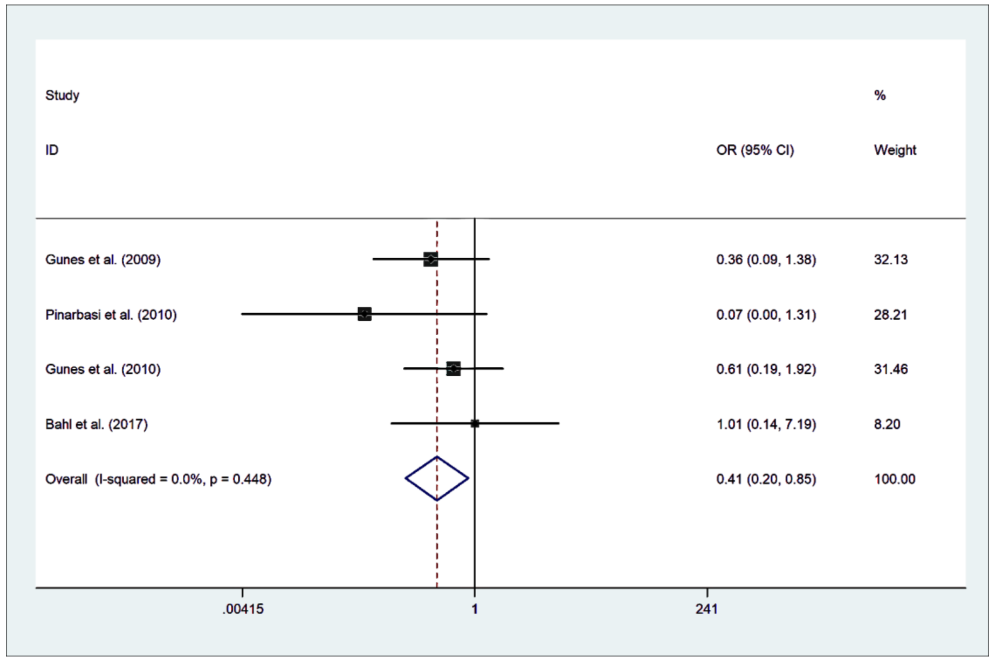

BB VS BA+AA
