# Supplementary material for: New insights of the correlation between AXIN2 polymorphism and cancer risk and susceptibility: evidence from 72 studies
Source: BMC Cancer. 2021 Apr 1;21:353. doi: 10.1186/s12885-021-08092-0 (PMC8017882; doi:10.1186/s12885-021-08092-0)
Supplement: Supplementary file 6 — Additional file 6 : Figure S5. Meta-analysis ofAXIN2-rs35415678 polymorphism and overall cancer risk in 3 genetic models. [file 12885_2021_8092_MOESM6_ESM.pdf]

Fig.S5 Meta-analysis of AXIN2-rs35415678 polymorphism and overall cancer risk in 3 genetic models.

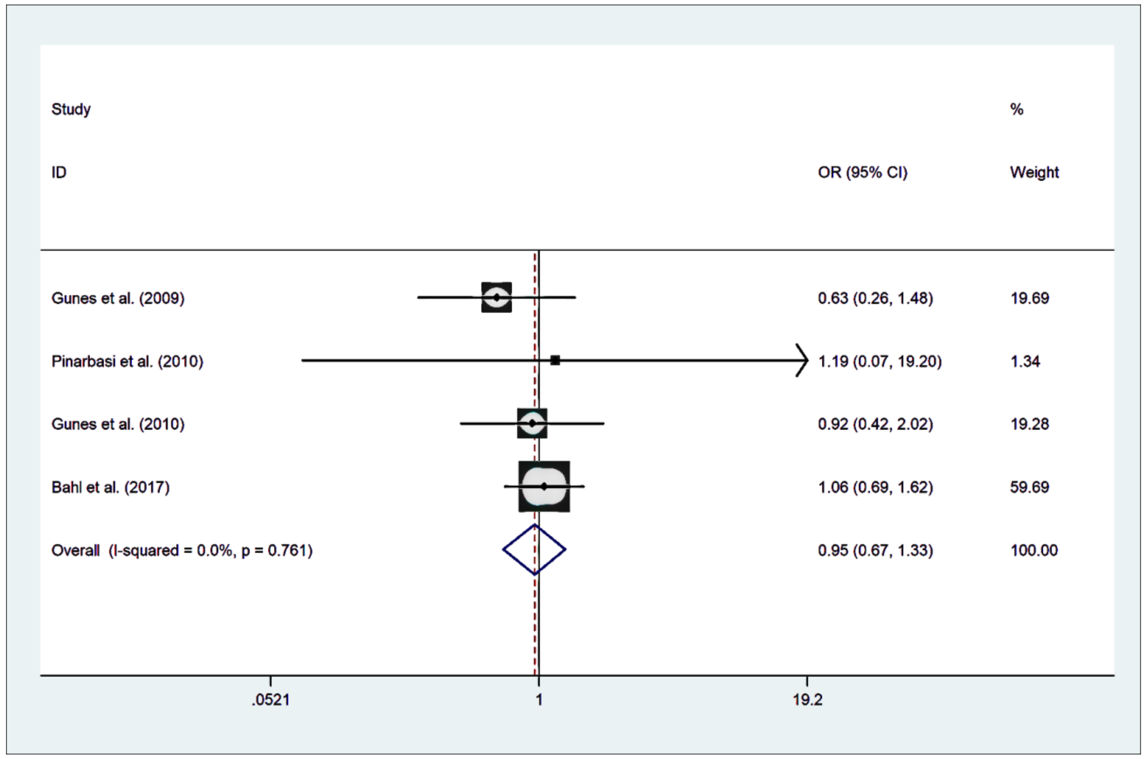

B VS A

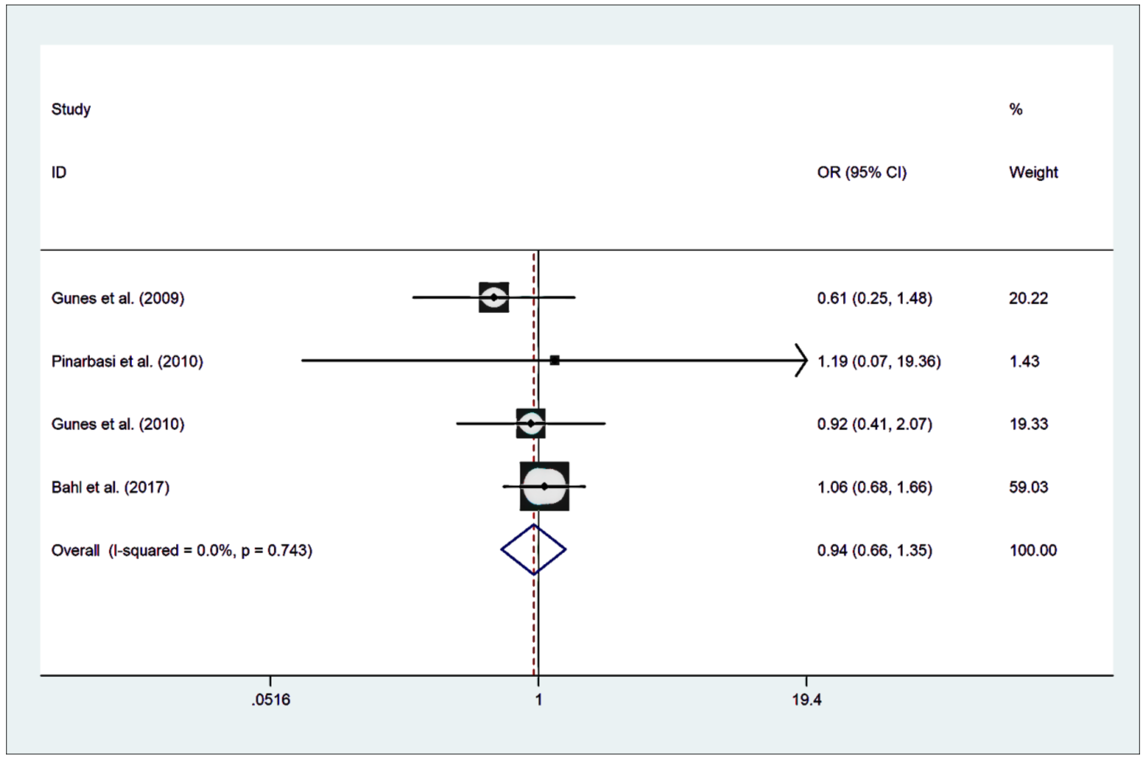

BA VS AA

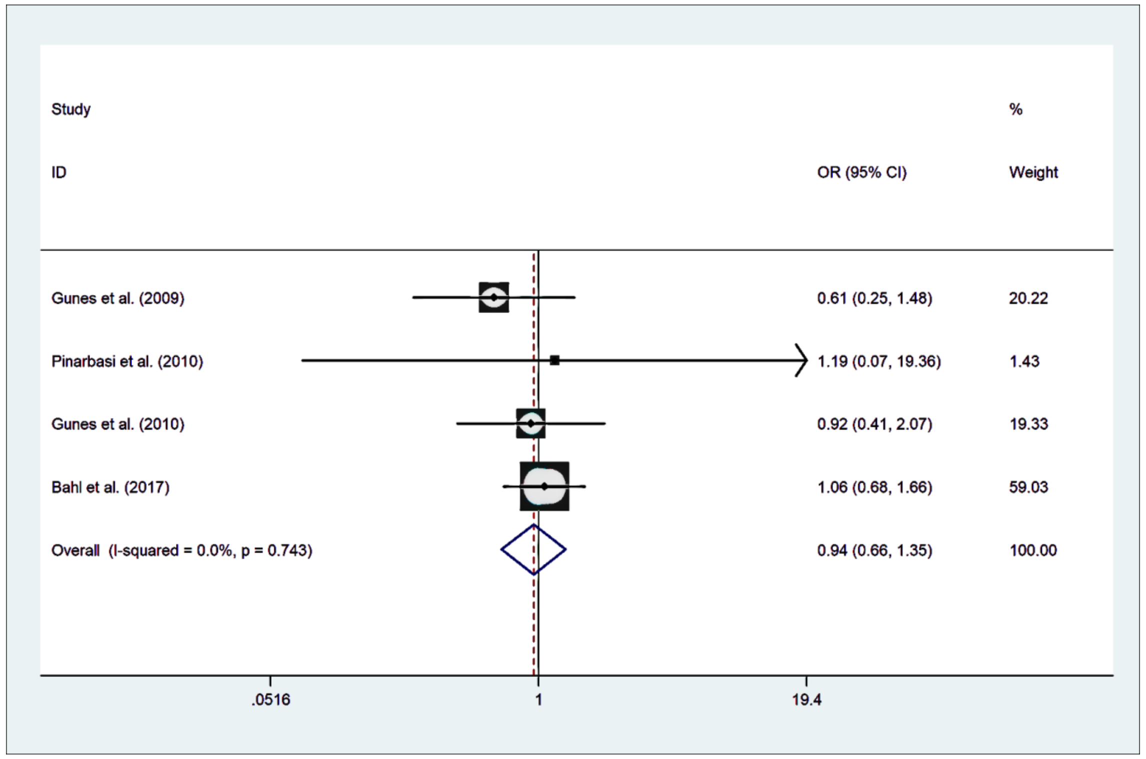

BB+BA VS AA
