# Supplementary material for: New insights of the correlation between AXIN2 polymorphism and cancer risk and susceptibility: evidence from 72 studies
Source: BMC Cancer. 2021 Apr 1;21:353. doi: 10.1186/s12885-021-08092-0 (PMC8017882; doi:10.1186/s12885-021-08092-0)
Supplement: Supplementary file 8 — Additional file 8 : Figure S7. Meta-analysis ofAXIN2-rs3923087 polymorphism and overall cancer risk in 5 genetic models. [file 12885_2021_8092_MOESM8_ESM.pdf]

# Fig.S7 Meta-analysis of AXIN2-rs3923087 polymorphism and overall cancer risk in 5 genetic models.

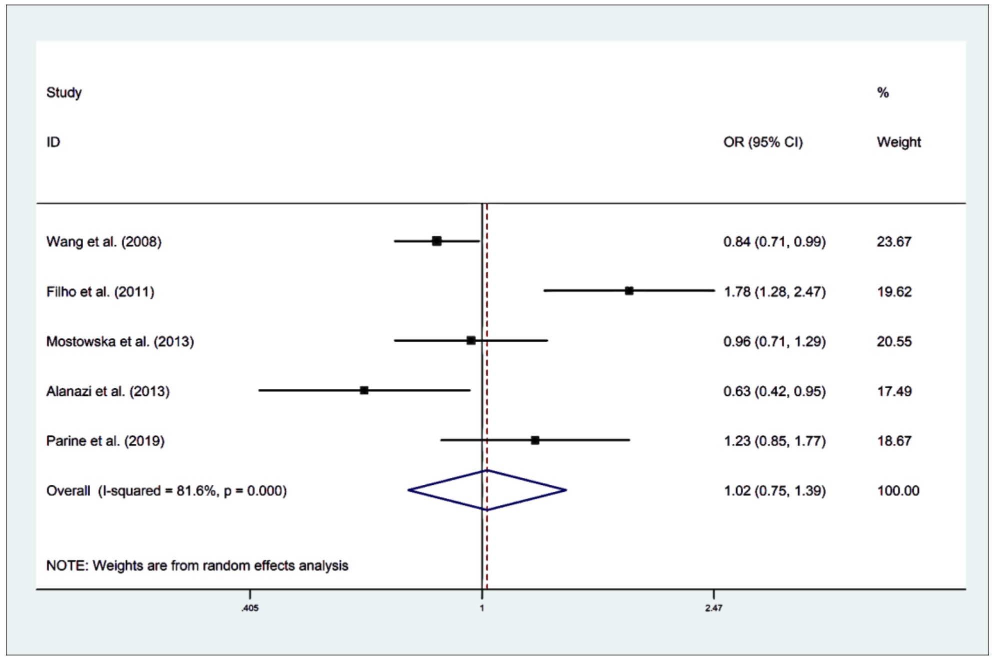


***B VS A***

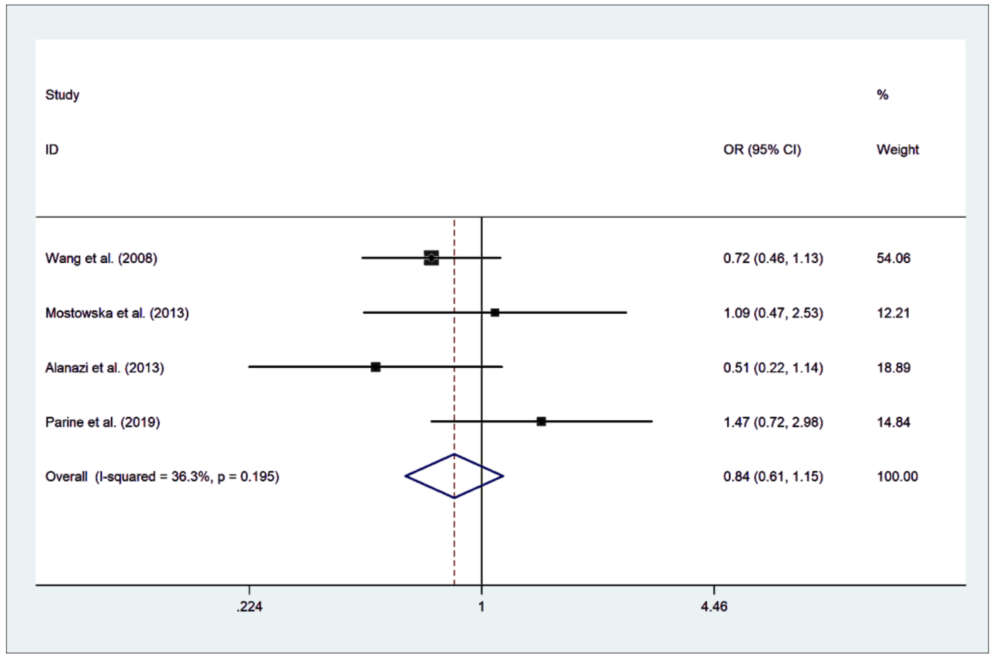


***BB VS AA***

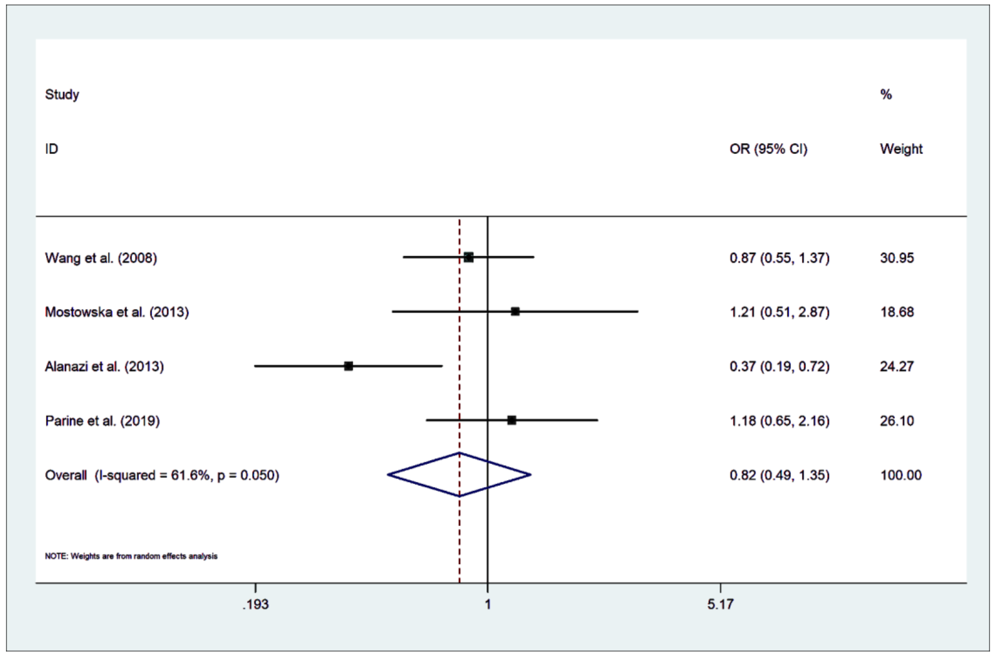


***BA VS AA***

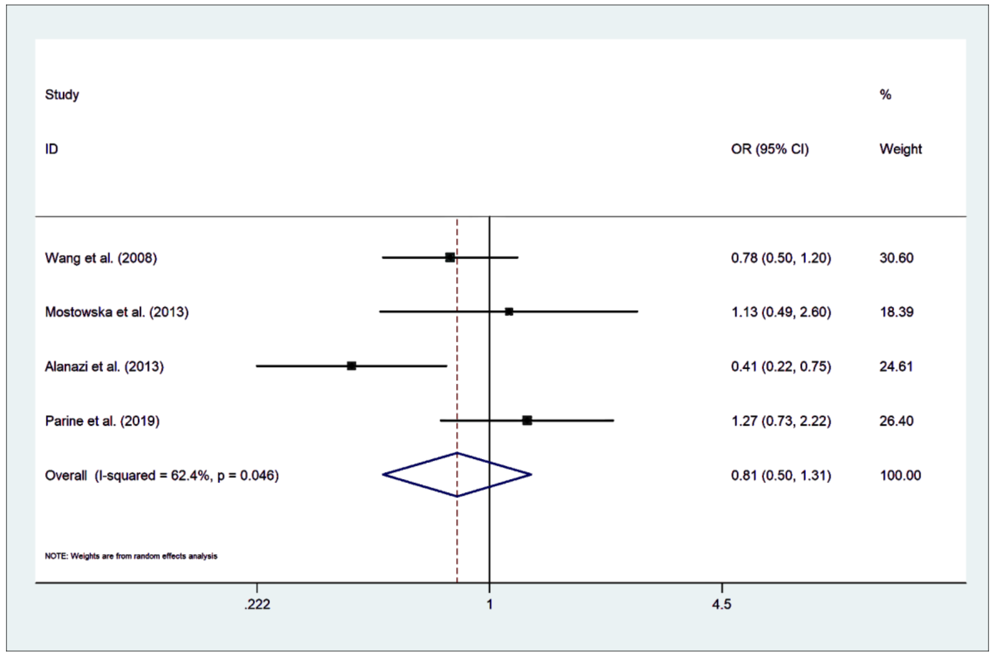


***BB+BA VS AA***

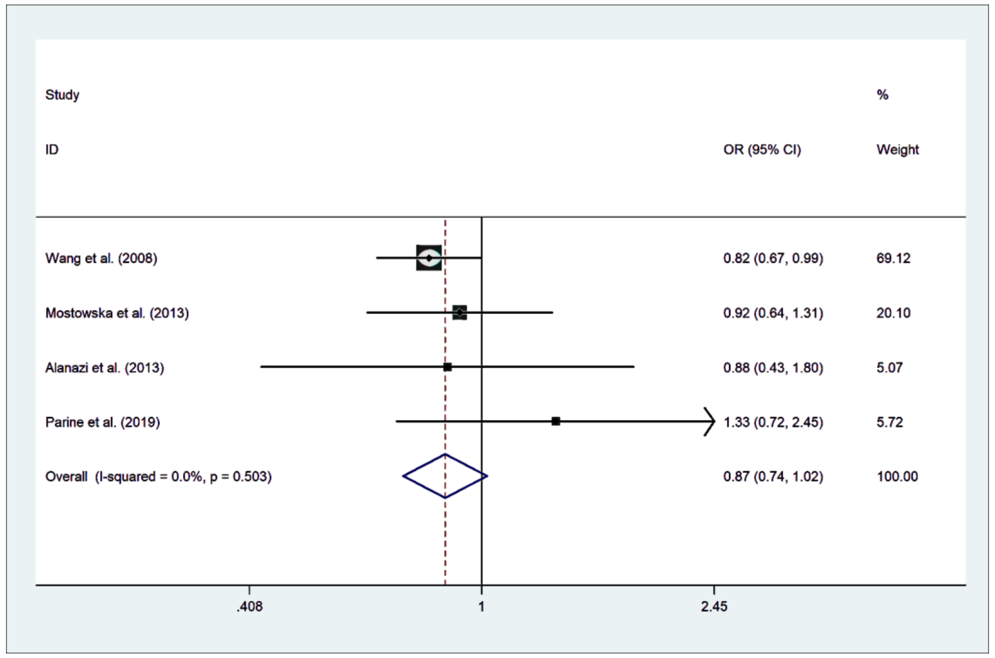


***BB VS BA+AA***
