# Supplementary figures and images for: New insights of the correlation between AXIN2 polymorphism and cancer risk and susceptibility: evidence from 72 studies
Source: BMC Cancer. 2021 Apr 1;21:353. doi: 10.1186/s12885-021-08092-0 (PMC8017882; doi:10.1186/s12885-021-08092-0)

Fig.S9 Meta-analysis of AXIN2-rs4791171 polymorphism and overall cancer risk in 5 genetic models.

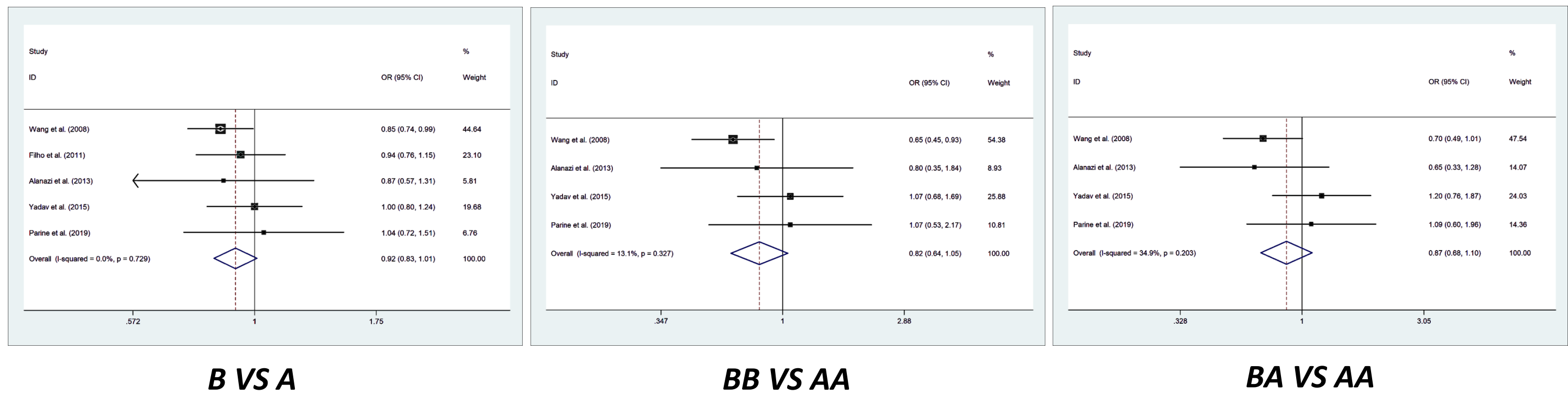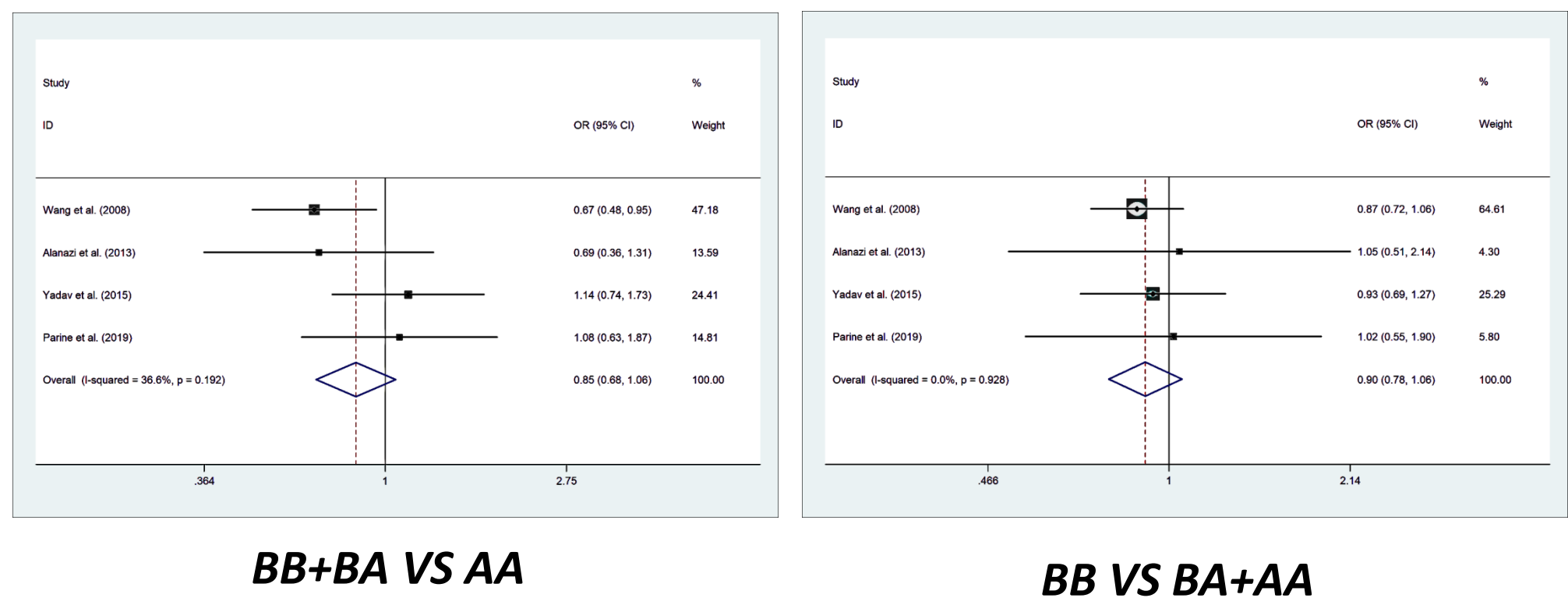

Supplement: Supplementary file 10 — Additional file 10 : Figure S9. Meta-analysis ofAXIN2-rs4791171 polymorphism and overall cancer risk in 5 genetic models. [file 12885_2021_8092_MOESM10_ESM.pdf]

Fig.S13 Sensitivity analysis of AXIN2 polymorphism and overall cancer ( B vs. A)

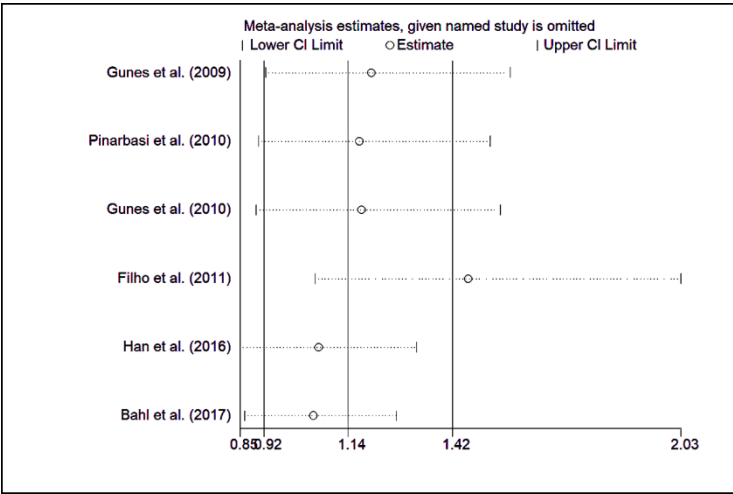

***rs2240307***

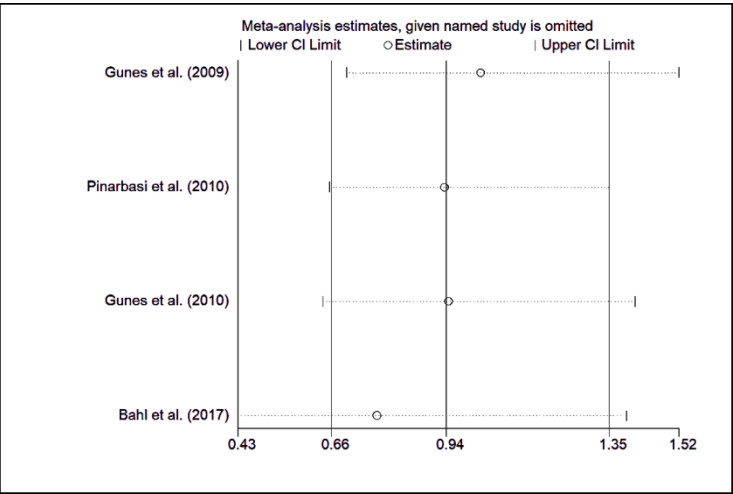

***rs35415678***

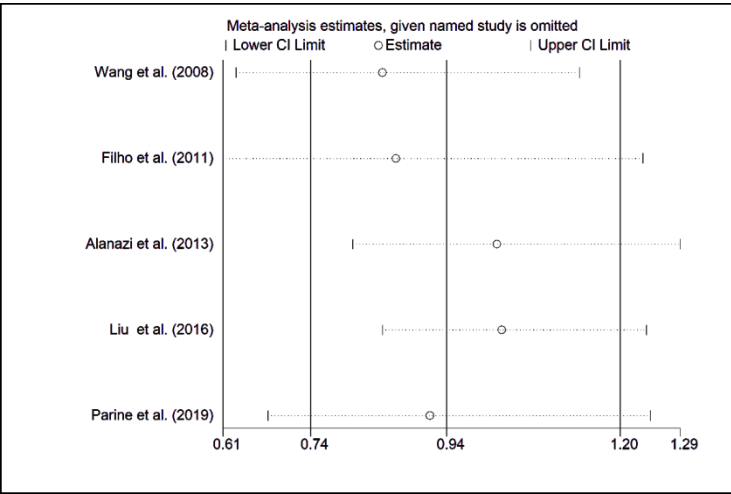

***rs3923086***

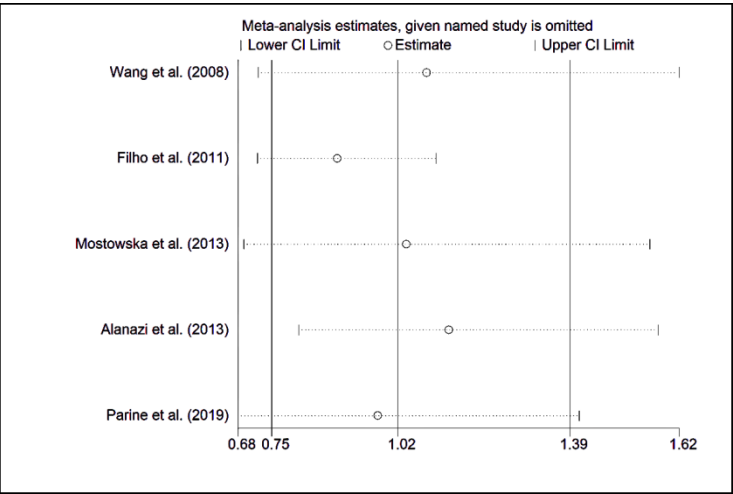

***rs3923087***

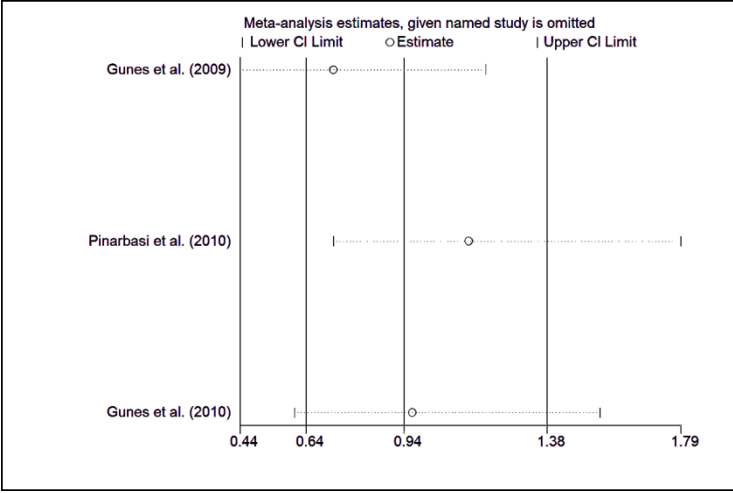

***rs4072245***

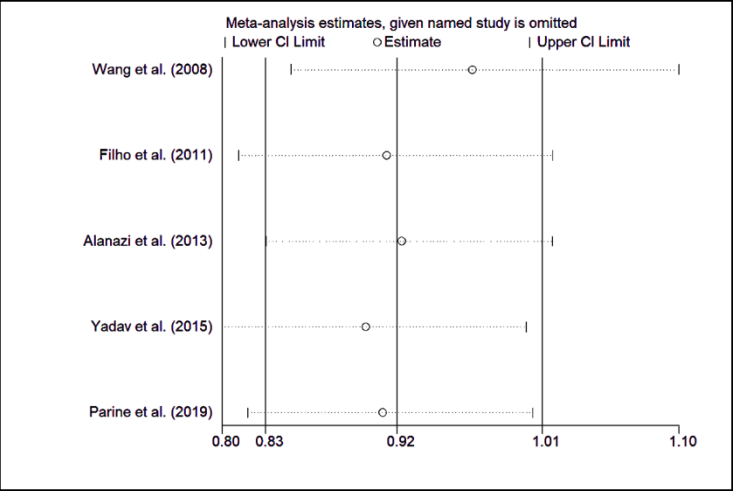

***rs4791171***

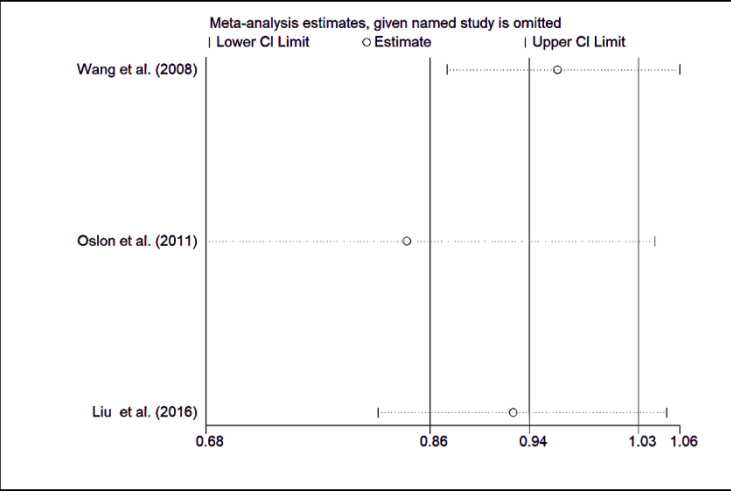

***rs7210356***

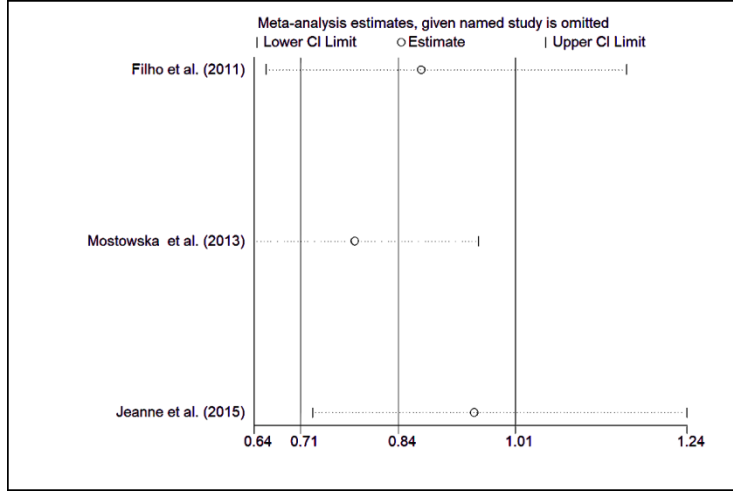

***rs7224837***

Supplement: Supplementary file 14 — Additional file 14 : Figure S13. Sensitivity analysis ofAXIN2 polymorphism and overall cancer (Bvs.A). The results of rs2240307, rs35415678, rs3923086, rs3923087, rs4072245, rs4791171, rs7210356, rs7224837 were presented in this figure. The dotted area represents the 95% confidence interval. [file 12885_2021_8092_MOESM14_ESM.pdf]

Fig.S14 Begg's plot of AXIN2 polymorphism and overall cancer ( B vs. A)

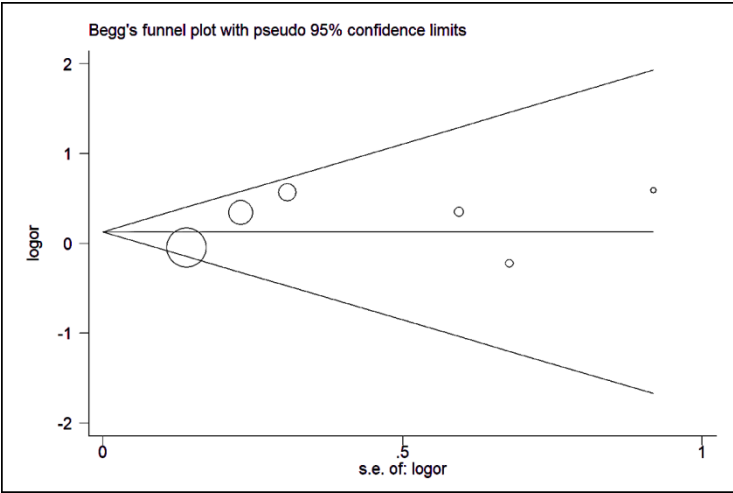

***rs2240307***

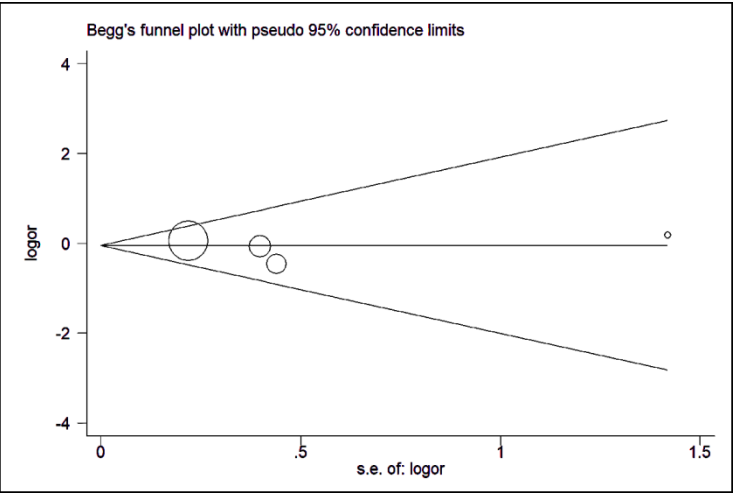

***rs35415678***

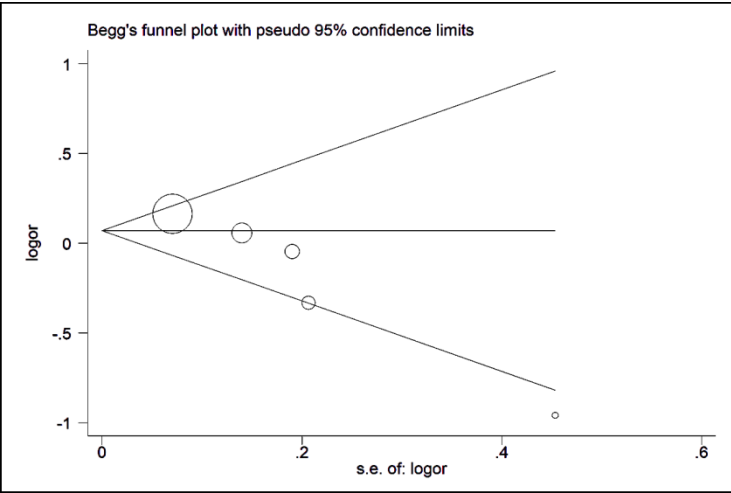

***rs3923086***

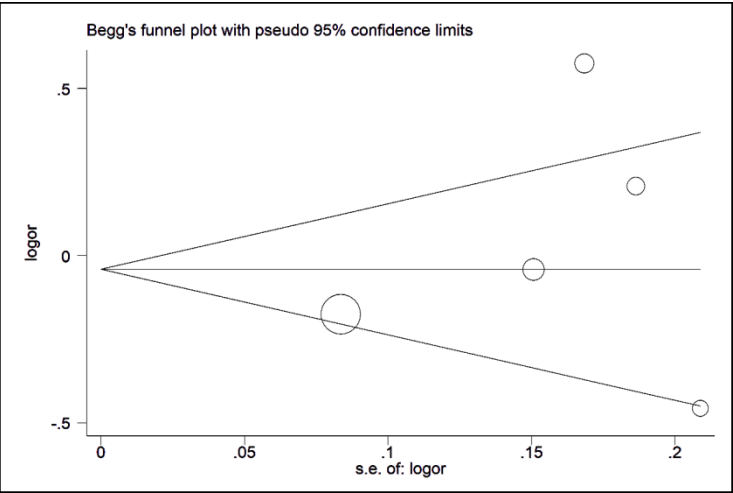

***rs3923087***

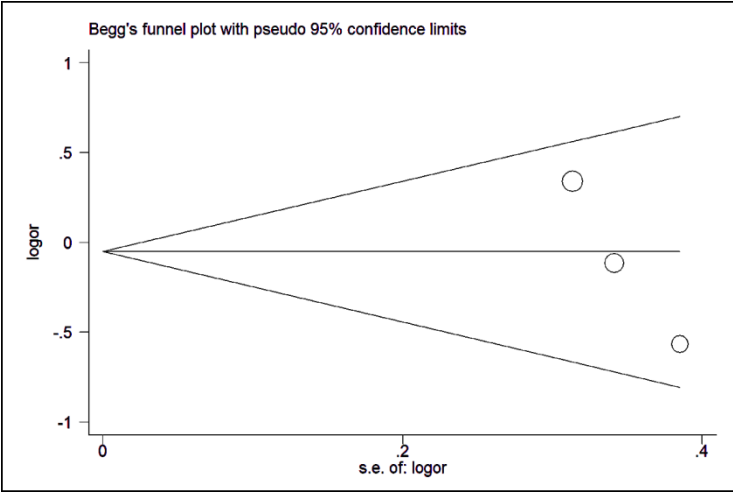

***rs4072245***

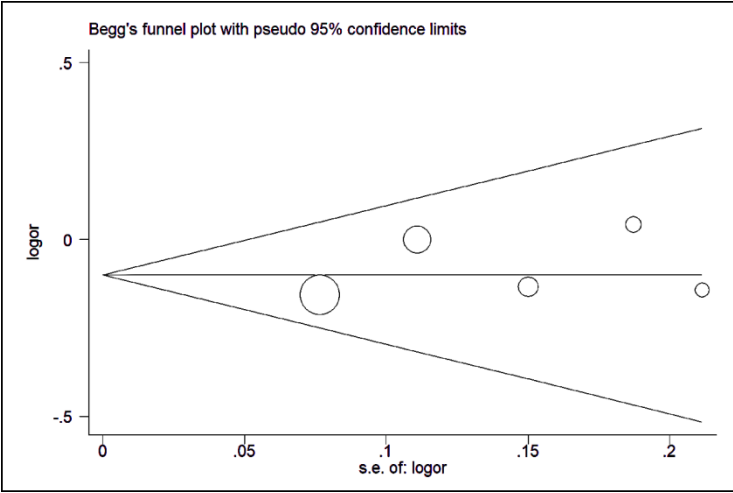

***rs4791171***

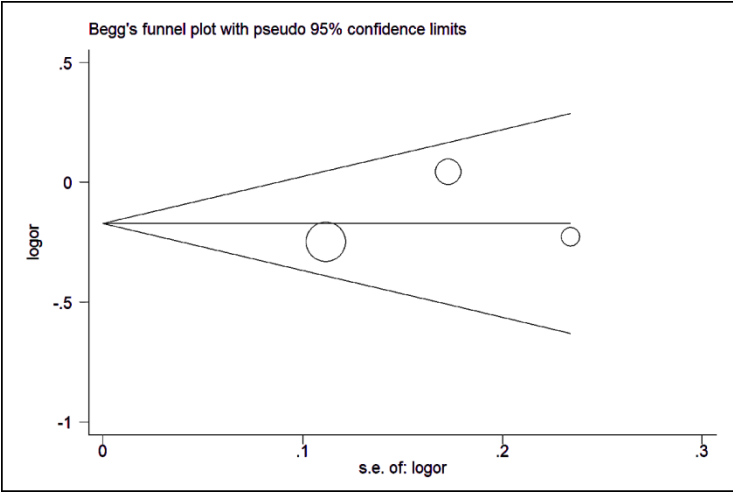

***rs7224837***

Supplement: Supplementary file 15 — Additional file 15 : Figure S14. Begg’splot ofAXIN2 polymorphism and overall cancer (Bvs.A). The results of rs2240307, rs35415678, rs3923086, rs3923087, rs4072245, rs4791171, rs7224837 were presented in this figure. The x-axis stands for the value of log (OR), and the y-axis stands for the value of natural logarithm of OR. The horizontal line stands for the overall estimated value of log (OR). The two diagonal lines in the figure represent the pseudo 95% confidence limits of the effect estimate. [file 12885_2021_8092_MOESM15_ESM.pdf]
